# Urine metabolomics signature reveals novel determinants of adrenal suppression in children taking inhaled corticosteroids to control asthma symptoms

**DOI:** 10.1002/iid3.1315

**Published:** 2024-07-19

**Authors:** Dung T. Tran, Yulu Chen, Yi Zheng, Julian Hecker, Daniel B. Hawcutt, Munir Pirmohamed, Jessica Lasky‐Su, Ann C. Wu, Kelan G. Tantisira, Michael J. McGeachie, Scott T. Weiss, Amber Dahlin

**Affiliations:** ^1^ Channing Division of Network Medicine Brigham and Women's Hospital and Harvard Medical School Boston Massachusetts USA; ^2^ University of Liverpool Liverpool UK; ^3^ Department of Population Medicine Harvard Medical School and Harvard Pilgrim Health Care Institute Boston Massachusetts USA; ^4^ Division of Pediatric Respiratory Medicine University of California San Diego and Rady Children's Hospital San Diego California USA

**Keywords:** adrenal function, asthma, biomarker, ICS, metabolomics

## Abstract

**Background:**

Asthma is routinely treated with inhaled corticosteroids (ICS). Asthma patients on ICS are at increased risk of adrenal suppression, a potentially serious effect of long‐term glucocorticoid exposure; however, this relationship is poorly understood. Therefore, this study aims to identify metabolite biomarkers related to adrenal suppression in asthma patients taking ICS.

**Methods:**

A total of 571 urine metabolites from 200 children with asthma on ICS in the Pharmacogenetics of Adrenal Suppression with Inhaled Steroids (PASS) cohort were profiled. Samples were grouped by peak plasma cortisol measurement as adrenal sufficient (>350 nmol/L) or insufficient (≤350 nmol/L) (outcome). Regression and discriminant‐based statistical models combined with network analyses were utilized to assess relationships between metabolites and the outcome. Finally, prioritized metabolites were validated using data from an ancillary study of the Childhood Asthma Management (CAMP) cohort with similar characteristics to PASS.

**Results:**

Ninety metabolites were significantly associated with adrenal suppression, of which 57 also could discriminate adrenal status. While 26 metabolites (primarily steroids) were present at lower levels in the adrenal insufficient patients, 14 were significantly elevated in this group; the top metabolite, mannitol/sorbitol, was previously associated with asthma exacerbations. Network analyses identified unique clusters of metabolites related to steroids, fatty acid oxidation, and nucleoside metabolism, respectively. Four metabolites including urocanic acid, acetylcarnitine, uracil, and sorbitol were validated in CAMP cohort for adrenal suppression.

**Conclusions:**

Urinary metabolites differ among asthma patients on ICS, by adrenal status. While steroid metabolites were reduced in patients with poor adrenal function, our findings also implicate previously unreported metabolites involved in amino acid, lipid, and nucleoside metabolism.

## Graphical abstract

Overall the study design of urinary metabolomics related to adrenal suppression in asthmatic children. This study profiled metabolomics of Pharmacogenetics of Adrenal Suppression with Inhaled Steroids (PASS) cohort including 200 children aged 5−18 years who were taking inhaled cortiscosteroids for asthma treatment. Significant metabolites were validated in Childhood Asthma Management (CAMP) cohort and previous studies. Pathway and network analysis were performed to understand the biochemical pathways associated with adrenal supppression.

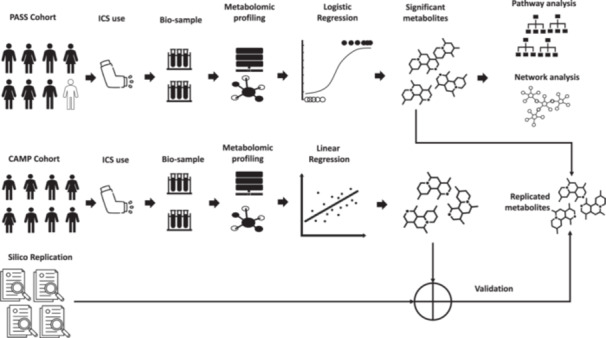

## 1 INTRODUCTION

Although considered safe and effective for asthma symptom control, the long‐term use of corticosteroids, both inhaled and oral, is associated with some significant risk of tertiary adrenal suppression (adrenal insufficiency), an important adverse event with the potential to lead to acute adrenal crisis due to suppression of endogenous cortisol production.^1^ While children with asthma who are taking inhaled corticosteroids (ICS) have lower risk of tertiary adrenal suppression than adults, they may be more susceptible to its effects as a result of their hypothalamic‐pituitary‐adrenal (HPA) axis development.^2^ The risk can be minimized through appropriate monitoring of adrenal function in high‐risk patients, and it is recommended that children using high doses of ICS undergo regular monitoring of HPA axis function.^2^, ^3^

Multiple additional risk factors contribute to tertiary adrenal suppression in children. These include the dose and duration of ICS use, and higher doses and longer duration of ICS exposure are associated with increased risk of adrenal suppression in children with asthma.^4^, ^5^, ^6^ Certain types of ICS (such as fluticasone propionate) may also be associated with a higher risk of adrenal suppression than others.^7^, ^8^, ^9^ In addition to ICS dose, type, and duration, the concomitant use of other medications that affect the metabolism of ICS is also a risk factor for adrenal suppression in children.^10^, ^11^, ^12^, ^13^ Finally, additional risk factors involved in increased adrenal suppression in children include younger age and lower body weight, genetic variation, and alteration of biochemical pathways related to steroid metabolism.^14^, ^15^ Due to genetic and environmental factors, 30% or more of asthma patients experience poor steroid responsiveness and require higher ICS doses to control asthma symptoms, putting these patients at increased risk of developing adrenal suppression.^16^ Both corticosteroid responsiveness and adrenal suppression demonstrate repeatable interindividual variation that is partly due to perturbation of genetic and molecular pathways^17^, ^18^, ^19^; however, the genetic and molecular contributions to both steroid response and adrenal suppression are not well understood.

Asthma metabolomic studies have also identified an increasing number of biochemical predictors of asthma risk and treatment responses to commonly prescribed medications, namely corticosteroids. Notably, metabolomics studies have found that the use of ICS in asthma was associated with alterations in amino acid, carbohydrate, and lipid metabolism, in sputum and sera.^18^, ^20^ A recently published a large scale metabolomic study with 14,000 individuals from four cohort studies revealed that patients with asthma who are treated with ICS are at risk of adrenal suppression.^19^ Plasma levels of cortisol were significantly decreased over 24‐h period in asthma patients with ICS treatment, compared with asthma patients without ICS.^19^ The study also identified 17 endogenous steroid metabolites which were significantly decreased in asthma patients. Of 17, DHEA‐S (dehydroepiandrosterone sulfate) and cortisol are biomarkers for adrenal suppression.^19^ While metabolomics represents a promising approach for investigating molecular drivers of adverse effects related to ICS use, the roles of specific metabolites related to these outcomes are not well understood. In this study, we investigated the hypothesis that unique metabolite signatures are associated with the development of tertiary adrenal suppression among children with asthma who are taking ICS. We evaluated this hypothesis through prospective untargeted metabolomics profiling combined with bioinformatics and statistical approaches to compare global metabolite profiles in urine samples from children with or without adrenal suppression.

## 2 METHODS

### 2.1 Description of samples and outcome

**Figure 1:**
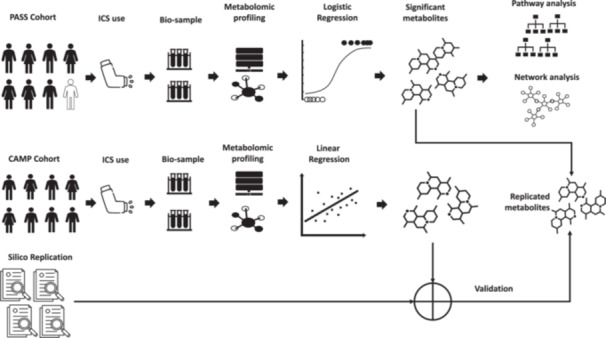
Metabolomics study design.

The study was conducted on urine samples collected from a subset of patients enrolled in the Pharmacogenetics of Adrenal Suppression with Inhaled Steroids (PASS) cohort.^17^ Participants were recruited into the PASS study from 25 sites across the United Kingdom and other relevant details of this cohort have been published.^17^, ^21^ In this study, we profiled samples from 200 non‐Hispanic white children of European descent aged 5−18 years, including 114 males and 86 females, who were taking ICS for asthma symptom control (described in Figure 1). The *primary outcome* was the presence of adrenal suppression diagnosed by low‐dose short Synacthen test, which was defined using a cutoff for peak plasma cortisol of less than 350 nmol/L; the clinical validity of this threshold in children with adrenal suppression was established.^17^ Samples were categorized into two groups based on peak plasma cortisol values to facilitate comparison of adrenal sufficient (>350 nmol/L) (*N* = 188) or insufficient (≤350 nmol/L) (*N* = 12) status (Figure 1). Urine samples were collected from all patients following the low‐dose short Synacthen test and were immediately stored at −80°C before shipping to Metabolon, Inc., for metabolite profiling.

### 2.2 Metabolomic analysis

Urinary metabolomic profiling was performed by Metabolon, Inc. Global untargeted profiling was conducted using Ultrahigh Performance Liquid Chromatography Tandem Mass Spectroscopy (UPLC‐MS/MS) with Thermo Scientific Q‐Exactive high resolution and accurate mass spectrometer. Batch variation was controlled in the analysis. The sample preparation and profiling methods were described in detail previously.^22^ Values for each urine sample were normalized by osmolality and each metabolite was median normalized to correct for analytical variation across runs. A total number of 984 named metabolites were identified by their mass to charge ratio (m/z), retention time (rt), and chromatographic data from all molecules present in the library using software developed at Metabolon, Inc. Metabolite pathways were annotated using the company in‐house software named Metabolon Pathway Analysis.

For quality control (QC), 815 of the 984 metabolites with measurements available in over 70% of patients were kept. In addition, the analysis excluded all named xenobiotics and partially characterized molecules. The final data set included 571 metabolite measurements from 200 patients (the list of metabolites is available by request). The data were processed with half the minimum imputation on the missing metabolite measurements, then log transformed and pareto scaled. All metabolites had a positive interquartile range (IQR) after QC. Similar QC pipelines have previously been applied in multiple peer‐reviewed publications.^18^, ^23^, ^24^

### 2.3 Statistical analysis

All statistical analyses were conducted using R statistical software version 4.2.2.^25^ Logistic regression was applied to quantify the relationship between peak plasma cortisol levels and urinary metabolite measurements (concentrations). The outcome variable in the logistic regression model was adrenal status (as sufficient (peak plasma cortisol≥350nmol/L) vs. insufficient (peak plasma cortisol<350nmol/L)). Since the distribution of the outcome variable in this data was heavily unbalanced by sample size between groups, which could introduce bias into the analysis, Synthetic Minority Over‐sampling Technique (SMOTE) was utilized to resample the data.^26^ In short, SMOTE resamples the unbalanced data by first randomly selecting an instance in the minority class as well as one instance among its *k* nearest neighbors. A new synthetic instance is then generated as a convex combination between the instance and the selected nearest neighbor. Synthetic data is added until the number of instances in the minority group is as desired. SMOTE is increasingly utilized in recent metabolomics investigations.^27^, ^28^, ^29^ While seeing increasingly more applications in metabolomics, SMOTE still raises concerns in terms of the quality of data that it generates and consequently, its accuracy in identifying significant metabolites. Therefore, we conducted a simulation to evaluate the quality of metabolites detected by SMOTE compared with regular Logistics regression as well as Saddle point Approximation models. This substudy is provided in the Supporting Information S1 section.

In this study, SMOTE was set with both *k* and the number of replications as three, so that the resampled data consisted of 188 adrenal sufficient and 48 adrenal insufficient patients. Following resampling of the data, logistic regression was performed to assess associations between predictor variables (individual metabolite measurements) and the outcome (adrenal status) and adjusting for gender and age. However, age was not significant in univariate or multivariate analyses, and was dropped in our final models. Logistic regression was utilized to evaluate the association between metabolites and outcomes (adrenal sufficient vs. insufficient). Mathematically, the model for each metabolite *i* is

$$P(y_{c}=\mathrm{Adrenalinsufficient})=\frac{1}{1+\text{exp}\left({-\beta }_{0}-\beta_{1}m_{i}-\beta_{2}g\right)}$$

where $y_{c}$ is the adrenal suppression diagnosis (adrenal sufficient vs insufficient); $m_{i}$ is the measurement for metabolite i; and g represents gender (female = 0, male = 1). To account for the randomness introduced by resampling, each model was fitted 1000 times, and the resulting *p* Values for each metabolite were then aggregated using their medians. *p* Values resulting from hypothesis testing were corrected using the false discovery rate (FDR), with a significance cutoff of 0.05.

Finally, Orthogonal Projections to Latent Structures Discriminant Analysis (OPLS‐DA) models were fitted on the resampled data using the *ropls* package in R.^30^ The models were also fitted 1000 times to account for randomness introduced through resampling. Q2Y metrics and 1000‐iteration permutation testing were used to avoid overfitting and to assess the statistical significance of the models. The priority of the metabolites was further determined with variable importance in projection (VIP) scores derived by median aggregation from the 1000 runs.

### 2.4 Pathway enrichment analysis

Pathway analysis of metabolites was first performed using MetaboAnalyst 5.0 (https://www.metaboanalyst.ca, accessed on 14 Feb 2023), specifying the hypergeometric test for over‐representation analysis and relative betweenness centrality for the pathway topology analysis.^31^ Of 571 metabolites, 289 metabolites with the Kyoto Encyclopedia of Genes and Genomes (KEGG) identifiers that also matched with molecules listed in the MetaboAnalyst database were input as the reference metabolome. All identified pathways were prioritized according to *p* Values from pathway enrichment analysis, with FDR adjusted *p* < .05 or unadjusted *p* < .05 representing cutoff values for enrichment significance.

Due to the poor representation of KEGG identifiers among our metabolite data, we also employed ChemRICH (https://chemrich.idsl.me/home, accessed on January 7, 2023), a statistical enrichment analysis based on chemical similarity instead of biochemical knowledge annotation.^32^ Out of 571 named metabolites, 470 were included in this analysis. ChemRICH clusters metabolites into nonoverlapping chemical groups using Tanimoto substructure chemical similarity coefficients and calculates cluster p‐values using the Kolmogorov−Smirnov test. The information required for this analysis included compound name, PubChem ID, the Simplified Molecular Input Line Entry System (SMILES) identity achieved from PubChem Database (https://pubchem.ncbi.nlm.nih.gov/idexchange/idexchange.cgi, accessed on January 7, 2023), unadjusted *p* Values from logistic regression, as well as fold changes (ratios of medians of adrenal insufficient group and that of adrenal sufficient group). The ChemRICH library requires input fold changes to be positive, therefore we evaluated the data without log transformation (as log‐transformed data may yield negative fold changes).

### 2.5 Network analysis

A correlation network was used to quantify and visualize the relationships among metabolites. Log‐transformed metabolite concentrations were used to calculate Pearson correlations of 90 significant metabolites using a threshold of 0.8 (*p* Value < 2.22 × 10^−16^). All computations for network visualization were generated in Python using the *network* package.^33^ Following correlation analysis, networks were then visualized using the MetScape plugin in Cytoscape 3.9.1.^34^, ^35^

### 2.6 Validation of metabolites

The Childhood Asthma Management Program (CAMP) is a randomized, placebo‐controlled, clinical trial of inhaled anti‐inflammatory treatments for mild to moderate childhood asthma.^36^ The CAMP cohort and study designed have been previously described.^36^ The validation group included 10 patients (aged 8−15 years) who underwent evaluation of HPA axis function at 36 months after receiving continuous treatment of budesonide (400 µg/day). After the HPA test, sera and urine samples were collected and cortisol measures were obtained over 24 h (baseline, 24‐h urine cortisol, and 30‐min and 60‐min serum cortisol measurements). Serum metabolites were profiled by the Broad Institute of MIT and Harvard (https://www.broadinstitute.org/).^19^, ^37^ Metabolites that overlapped in PASS and CAMP were investigated to determine whether they met significant thresholds in both cohorts. After QC procedures (as described above), 482 metabolites were included in linear regression analysis, performed as described in the previous section. Linear regression models were fitted with the outcome variable specified as either serum cortisol measurement (baseline, 30 and 60‐min serum cortisol measurements) or the 24‐h urinary cortisol measurement, and predictor variables as each of the metabolite measurements individually, adjusted for gender and age.

## 3 RESULTS

### 3.1 Study cohort and metabolomics profiling results

**Figure 2:**
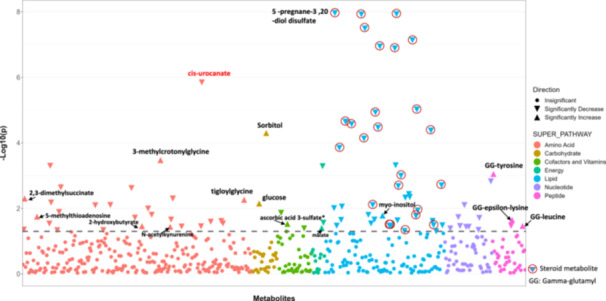
Manhattan plot of *p* Values from 571 metabolites in logistic regression models.

Following metabolite profiling and QC procedures, 571 metabolites were identified in 200 urine samples from PASS participants. The greatest proportion of metabolites was categorized as amino acids (45.7%), followed by lipids (23.8%), with the remainder as carbohydrates (4.90%), cofactors and vitamins (6.83%), energy cycle (2.63%), nucleotide (9.46%), peptide (6.65%) (Figure 2 and Figure S1a).

**Figure 3:**
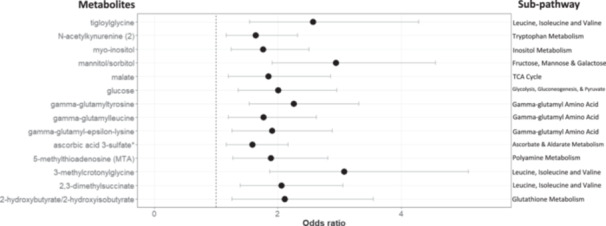
Urinary metabolites with significantly elevated levels in adrenal insufficient patients. The dotted line indicates the cutoff for OR. OR and 95% CIs are shown for each metabolite. Statistical significance was determined using an adjusted FDR < 0.05. CI, confidence interval; FDR, false discovery rate; OR, odds ratio;

Of the 571 metabolites, logistic regression models identified 90 (15.8%) metabolites that were significantly associated with adrenal status (FDR < 0.05) (Figure 2); statistical and pathway information are provided in the Table S1. Reflecting the overall composition of the measured metabolites, these were primarily lipids (45.6%), amino acids (31.1%) and nucleotides (10.0%) Figure S1b). Twenty‐six of the 90 significantly associated metabolites were annotated to steroid sub‐pathways, including androgenic steroids (13), pregnenolone (4), progestin steroids (2) and corticosteroids (7) (Table 1). All 26 steroid metabolites were significantly reduced in the adrenal insufficient samples compared to adrenal sufficient (Table 1 and Figure 2). Nine of the 26 steroid metabolites were included among the top 10 most significant metabolites, with the most significant metabolite, 5α‐pregnane‐3β,20α‐diol disulfate, belonging to the progestin steroids sub‐pathway (Table 1 and Figure 2). Notably, of the top 10 metabolites significantly associated with adrenal suppression, one non‐steroid metabolite, cis‐urocanate, was ranked as 8th by *p* Value and was reduced in the adrenal insufficient samples (odds ratio [OR] = 0.20 [95% confidence interval [CI] 0.12−0.35]; *p* = 1.42 × 10^−6^) (Figure 2 and Table S1). Moreover, 14 metabolites were significantly associated with increased levels in the adrenal insufficient group (Figure 3). Six of these 14 were amino acid metabolites, three were peptides, and two were carbohydrates (Figure 3). The peptides, γ‐glutamyl leucine, γ‐glutamyl tyrosine, and γ‐glutamyl epsilon lysine, were annotated to the γ‐glutamyl amino acid pathway. Among the amino acids, three were annotated to the leucine, isoleucine, and valine metabolism pathways, while the two carbohydrates included glucose and mannitol/sorbitol (Figure 3). Based on FDR adjusted *p‐value*, mannitol/sorbitol was the most significant of the 14 metabolites found at higher levels in the adrenal insufficient group (*p* = 4.97 × 10^−5^); the second was 3‐methylcrotonylglycine (*p* = 3.33 × 10^−4^) (Figure 3).

**Table 1:** Steroid metabolites significantly associated with adrenal suppression in the PASS cohort.

Metabolite	Metabolite sub‐pathway	OR (95% CI)	*p* Value^a^	Study
5 alpha‐pregnan‐3beta,20alpha‐diol disulfate	Progestin steroids	0.25 (0.16, 0.37)	1.10E‐08	[19]
21‐hydroxypregnenolone disulfate	Pregnenolone steroids	0.22 (0.14, 0.34)	1.18E‐08	[19]
Andro steroid monosulfate C19H28O6S^a,^*	Androgenic steroids	0.3 (0.21, 0.43)	1.21E‐08	[19]
Pregnen‐diol disulfate*	Pregnenolone steroids	0.28 (0.19, 0.41)	3.22E‐08	[19]
Pregnenetriol disulfate*	Pregnenolone steroids	0.29 (0.2, 0.43)	7.32E‐08	[19]
Androstenediol (3beta,17beta) disulfate^b^	Androgenic steroids	0.32 (0.22, 0.46)	1.14E‐07	[19]
16 a‐hydroxy DHEA 3‐sulfate	Androgenic steroids	0.39 (0.29, 0.53)	1.31E‐07	[19]
Androsterone glucuronide	Androgenic steroids	0.41 (0.29, 0.57)	9.60E‐06	[19]
Dehydroandrosterone glucuronide	Androgenic steroids	0.43 (0.31, 0.59)	1.18E‐05	
Androstenediol (3beta,17beta) disulfate^a^	Androgenic steroids	0.46 (0.34, 0.62)	2.26E‐05	[19]
Pregnanediol‐3‐glucuronide	Progestin steroids	0.44 (0.32, 0.61)	2.69E‐05	[19]
17 alpha‐hydroxypregnanolone glucuronide	Pregnenolone steroids	0.53 (0.41, 0.68)	3.43E‐05	[38]
Epiandrosterone glucuronide	Androgenic steroids	0.47 (0.35, 0.64)	4.18E‐05	
11 beta‐hydroxyandrosterone glucuronide	Androgenic steroids	0.37 (0.25, 0.56)	7.38E‐05	[19]
Dehydroepiandrosterone sulfate (DHEA‐S)	Androgenic steroids	0.53 (0.4, 0.69)	1.42E‐04	[19]
Epiandrosterone sulfate	Androgenic Steroids	0.57 (0.43, 0.74)	1.01E‐03	[19]
3 alpha,21‐dihydroxy‐5beta‐pregnane‐11,20‐dione 21‐glucuronide	Corticosteroids	0.56 (0.42, 0.75)	1.98E‐03	
11‐dehydrocorticosterone sulfate	Corticosteroids	0.57 (0.43, 0.76)	2.10E‐03	
11 beta‐hydroxyandrosterone sulfate^b^	Androgenic steroids	0.6 (0.45, 0.8)	7.88E‐03	
Cortisol 21‐sulfate	Corticosteroids	0.62 (0.47, 0.82)	1.16E‐02	
Etiocholanolone glucuronide	Androgenic steroids	0.68 (0.53, 0.86)	1.57E‐02	[19]
Cortolone glucuronide^a^	Corticosteroids	0.61 (0.44, 0.84)	2.32E‐02	[20]
Cortolone glucuronide^b^	Corticosteroids	0.62 (0.46, 0.85)	2.40E‐02	[20]
Tetrahydrocortisol	Corticosteroids	0.63 (0.46, 0.86)	2.89E‐02	[19]
11‐ketoetiocholanolone sulfate	Androgenic steroids	0.65 (0.48, 0.87)	3.25E‐02	
Cortisone	Corticosteroids	0.65 (0.47, 0.89)	5.00E‐02	[18]

Abbreviation: CI, confidence interval; FDR, false discovery rate; OR, odds ratio; PASS, Pharmacogenetics of Adrenal Suppression with Inhaled Steroids.

^a^ Metabolite names with an asterisk (*) indicate that the identity has not been confirmed based on analytical standard;

^b^ FDR < 0.05.

Overall, OPLS‐DA models were able to clearly separate the two adrenal status groups. In total, 57 metabolites were potential predictors of adrenal suppression (variable importance scores [VIP] > 1.5) (Table S1). Of these, 24 metabolites were annotated to major steroid hormone biosynthesis sub pathways. The top‐ranked metabolite was 21‐hydroxypregnenolone disulfate, a steroid metabolite (VIP: 3.82). Six nonsteroid metabolites present among the top 20 (VIP > 2.08) included cis‐urocanate, mannitol/sorbitol, 3‐methylcrotonylglycine, 1‐methylguanine, (S)−3‐hydroxybutyrylcarnitine, and N‐acetyl‐cadaverine (Figure S2). The remaining metabolite, glycoursodeoxycholate, approached significance with a *p* value of 0.06. Furthermore, 56 of the 57 metabolites were also significantly associated with adrenal suppression in the logistic regression models, and 33 significant metabolites from logistic models still achieved VIP values over 1.1 (Table S1).

### 3.2 Enrichment of biological and biochemical pathways related to adrenal suppression

Thirty four of 90 significant metabolites associated with adrenal suppression by logistic regression models, and with KEGG identifiers matching those in the Metaboanalyst 5.0 database, were analyzed for pathway enrichment. Twenty‐three perturbed metabolic pathways were identified, among which the steroid hormone biosynthesis pathway was the most influential in adrenal insufficient samples compared with adrenal sufficient, with an FDR adjusted *p* value of 2.38 × 10^−4^ (Table S2). Due to lacking KEGG IDs, only six hit metabolites could be matched in the steroid hormone metabolism pathway while there were 26 total steroid metabolites significantly associated with adrenal insufficiency, based on logistic regression models. The second most altered metabolic pathway (unadjusted *p* = 9.06 × 10^−3^) was galactose metabolism which included three hit metabolites: glucose, mannitol/sorbitol, and myo‐inositol (Table S2).

Based on chemical similarity provided by ChemRICH, the output revealed 22 significantly enriched metabolite clusters with FDR adjusted *p* value less than 0.05 (Table 2 and Figure S3). The list of metabolites in each cluster was listed in Table S3. The top three most significantly altered metabolite clusters included androgenic steroids, pyrimidine metabolism, and fatty acid metabolism (Table 2). Of the 22 clusters, 10 contained only metabolites that were decreased in adrenal insufficient group (annotated as blue clusters in Figure S3). Four of these 10 clusters represented lipid metabolites, and included androgenic steroids, corticosteroids, fatty acids (branched‐chain amino acid metabolism), and members of the dicarboxylate metabolism pathway. Two clusters (histidine and creatine metabolism, respectively) included only amino acids. The remaining clusters were represented by purine metabolism (nucleotides) and amino sugar metabolism (carbohydrates). Furthermore, γ‐glutamyl amino acid was the only cluster containing all seven metabolites that were enriched in the adrenal insufficient group, suggesting a dysfunction in peptide metabolism (annotated as red cluster in Figure S3).

**Table 2:** Metabolite sub‐pathways differing between adrenal sufficient and insufficient groups.

Cluster name^a^	Cluster size^b^	FDR *p* Value	Key compound	No of increased metabolites^c^	No of decreased metabolites^c^
Androgenic steroids	7	9.90E‐19	Androstenediol (3beta,17beta) disulfate (2)	0	7
Pyrimidine metabolism, uracil containing	12	2.80E‐05	Uracil	1	7
Fatty acid metabolism (also BCAA metabolism)	3	7.20E‐05	Propionylcarnitine (C3)	0	3
Histidine metabolism	23	1.00E‐04	Cis‐urocanate	0	11
Lysine metabolism	20	1.80E‐04	N‐acetyl‐cadaverine	2	5
G‐glutamyl amino acid	10	4.30E‐04	g‐glutamyltyrosine	7	0
Purine metabolism, guanine containing	3	1.50E‐03	1‐methylguanine	0	2
Secondary bile acid metabolism	9	1.50E‐03	12‐dehydrocholate	1	3
Aminosugar metabolism	8	1.60E‐03	N‐acetylglucosaminitol	0	1
Leucine, isoleucine and valine metabolism	35	3.70E‐03	3‐methylcrotonylglycine	4	6
Polyamine metabolism	10	7.30E‐03	5‐methylthioadenosine (MTA)	1	4
Fatty acid, dicarboxylate	17	8.50E‐03	4‐octenedioate	0	2
Pyrimidine metabolism, cytidine containing	5	1.40E‐02	3‐methylcytidine	1	2
Corticosteroids	4	1.40E‐02	Cortisol 21‐sulfate	0	3
Tryptophan metabolism	27	2.20E‐02	Xanthurenate	2	7
Methionine, cysteine, SAM, and taurine metabolism	20	2.70E‐02	Taurine	2	2
Purine metabolism, (hypo)xanthine/inosine containing	9	2.70E‐02	1‐methylhypoxanthine	0	4
Tyrosine metabolism	26	2.70E‐02	3,4‐dihydroxyphenylacetate	2	6
TCA cycle	13	2.70E‐02	2‐methylcitrate	1	1
Dipeptide	8	2.70E‐02	phenylalanylglycine	0	2
Acetylated peptides	10	2.70E‐02	phenylacetylglutamine	1	0
Creatine metabolism	4	4.20E‐02	guanidinoacetate	0	1

Abbreviation: BCAA, branched‐chain amino acid.

^a^ Cluster name are sub pathways which are annotated by Metabolon Inc.;

^b^ Cluster size is number of metabolites in each cluster;

^c^ Increased refers to elevated concentrations of metabolites in the adrenal insufficient group, while decreased indicates reduced concentrations in the adrenal insufficient group. Increased or decreased levels are determined based on fold change, calculated as the ratio of the medians of imputed metabolite measurements in adrenal insufficient vs adrenal sufficient patients; a ratio value < 1 indicates lower levels of metabolites in adrenal insufficient compared to adrenal sufficient groups.

### 3.3 Network analysis of metabolic interaction

**Figure 4:**
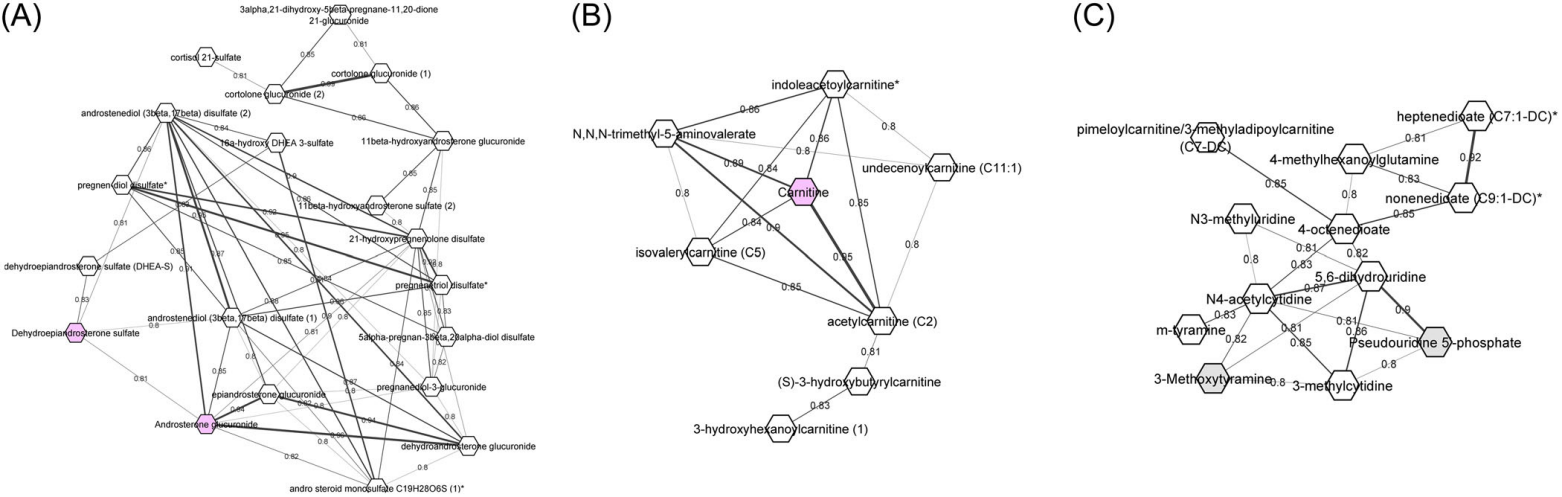
Network visualization of Pearson correlations using log transformed metabolite concentrations with absolute values higher than 0.8. (A) Cluster 1 includes 20 steroid metabolites. (B) Cluster 2 is comprised of eight metabolites from fatty acid metabolism, and carnitine, trytophan and leucine metabolism pathways. (C) Cluster 3 includes 12 metabolites from fatty acid metabolism and nucleotide metabolism. Nodes (circles) represent individual metabolites. Thickness of the edges (lines connecting nodes) represents the strength of correlation while the number label of the edges are values for Pearson correlations. Node width represents strength of correlation. Pink nodes represent metabolites with KEGG IDs. KEGG, Kyoto Encyclopedia of Genes and Genomes.

The metabolite correlation network of 90 significant metabolites was mainly composed of three clusters that included a steroid metabolite cluster and two non‐steroid metabolite clusters (Figure 4). The largest cluster was the steroid biosynthesis pathway and included 20 metabolites with significantly lower concentrations in adrenal insufficient samples (Table 1 and Figure 4A). The second cluster comprised eight compounds related to carnitine metabolism: indoleacetoylcarnitine*, isovalerylcarnitine (C5), N,N,N‐trimethyl‐5‐aminovalerate, undecenoylcarnitine (C11:1), carnitine, acetylcarnitine (C2), (S)‐3‐hydroxybutyrylcarnitine and 3‐hydroxyhexanoylcarnitine (1) (Table 3 and Figure 4B). Of these eight, five metabolites were significantly decreased in the adrenal insufficient group, based on ChemRICH (Table 3). The remaining three did not have SMILES identifiers and were not included in the ChemRICH analysis. Based on ORs from logistic regression, these three metabolites showed lower levels in adrenal insufficient samples compared with adrenal sufficient samples (Table 3). The third cluster included 12 compounds: lipids (4‐methylhexanoylglutamine, nonenedioate (C9:1‐DC) *, 4‐octenedioate, pimeloylcarnitine/3‐methyladipoylcarnitine (C7‐DC), heptenedioate (C7:1‐DC) *); amino acids (3‐methoxytyramine, m‐tyramine); and nucleotides (3‐methylcytidine, N3‐methyluridine, N4‐acetylcytidine, 5,6‐dihydrouridine, pseudouridine) (Table 3 and Figure 4C). Five nucleotide compounds belonged to pyrimidine metabolism, two amino acids compounds belonged to tyrosine metabolism, and five lipids belonged to fatty acid metabolism pathways (Table 3). Eight of 12 compounds showed decreased amount in adrenal insufficient groups based on ChemRICH analysis (Table 3). Four compounds did not have SMILES identifiers, but based on ORs, all four had ORs less than 1.00, demonstrating lower levels in the adrenal insufficient groups (Table S1).

**Table 3:** Clusters of highly correlated nonsteroid urinary metabolites identified by network analysis.

Metabolite	Metabolite super‐pathway^a^	Metabolite sub‐pathway^a^	Effect direction (ChemRICH)	OR (95% CI)^b^	FDR *P*‐value^b^
Cluster 2 (Figure 4B)					
Carnitine	Lipid	Carnitine Metabolism	down	0.61 (0.44, 0.84)	2.20E‐02
(S)‐3‐hydroxybutyrylcarnitine	Lipid	Fatty Acid Metabolism (Acyl Carnitine, Hydroxy)	down	0.47 (0.33, 0.66)	4.85E‐04
3‐hydroxyhexanoylcarnitine (1)	Lipid	Fatty Acid Metabolism (Acyl Carnitine, Hydroxy)	N/A^c^	0.6 (0.42, 0.86)	3.41E‐02
Undecenoylcarnitine (C11:1)	Lipid	Fatty Acid Metabolism (Acyl Carnitine, Monounsaturated)	N/A^c^	0.6 (0.45, 0.79)	4.51E‐03
Acetylcarnitine (C2)	Lipid	Fatty Acid Metabolism (Acyl Carnitine, Short Chain)	down	0.6 (0.44, 0.81)	9.79E‐03
Isovalerylcarnitine (C5)	Amino Acid	Leucine, Isoleucine and Valine Metabolism	down	0.62 (0.46, 0.84)	1.92E‐02
N,N,N‐trimethyl‐5‐aminovalerate	Amino Acid	Lysine Metabolism	down	0.54 (0.39, 0.76)	5.03E‐03
Indoleacetoylcarnitine*	Amino Acid	Tryptophan Metabolism	N/A^c^	0.63 (0.49, 0.82)	8.00E‐03
Cluster 3 (Figure 4C)					
pimeloylcarnitine/3‐methyladipoylcarnitine (C7‐DC)	Lipid	Fatty Acid Metabolism (Acyl Carnitine, Dicarboxylate)	N/A^c^	0.48 (0.32, 0.73)	8.80E‐03
4‐methylhexanoylglutamine	Lipid	Fatty Acid Metabolism (Acyl Glutamine)	N/A^c^	0.62 (0.44, 0.86)	3.32E‐02
nonenedioate (C9:1‐DC)*	Lipid	Fatty Acid, Dicarboxylate	N/A^c^	0.49 (0.33, 0.71)	3.69E‐03
heptenedioate (C7:1‐DC)*	Lipid	Fatty Acid, Dicarboxylate	N/A^c^	0.47 (0.31, 0.71)	4.69E‐03
4‐octenedioate	Lipid	Fatty Acid, Dicarboxylate	down	0.53 (0.35, 0.79)	1.72E‐02
3‐methylcytidine	Nucleotide	Pyrimidine Metabolism, Cytidine containing	down	0.5 (0.31, 0.81)	3.64E‐02
N4‐acetylcytidine	Nucleotide	Pyrimidine Metabolism, Cytidine containing	down	0.54 (0.35, 0.84)	4.10E‐02
5,6‐dihydrouridine	Nucleotide	Pyrimidine Metabolism, Uracil containing	down	0.43 (0.25, 0.73)	1.85E‐02
N3‐methyluridine	Nucleotide	Pyrimidine Metabolism, Uracil containing	down	0.62 (0.44, 0.86)	3.46E‐02
Pseudouridine	Nucleotide	Pyrimidine Metabolism, Uracil containing	down	0.52 (0.33, 0.82)	3.67E‐02
3‐methoxytyramine	Amino Acid	Tyrosine Metabolism	down	0.52 (0.34, 0.8)	2.56E‐02
m‐tyramine	Amino Acid	Tyrosine Metabolism	down	0.56 (0.38, 0.82)	2.87E‐02

^a^ Super pathway and sub pathway were annotated by Metabolon Inc.

^b^ OR and FDR p‐values were from logistic regression models.

^c^ N/A means no information due to no SMILES and PubChem ID input for ChemRICH analysis.

To identify which nodes within the network could be of particular relevance to the structure of the network, we estimated the connectivity and neighborhood density properties of all network nodes. Our findings showed that network hubs, i.e., the nodes with a high level of connectivity based upon the number of edges, included androstenediol (3beta, 17beta) disulfate (2) with 10 edges, androstenediol (3beta, 17beta) disulfate (1), and 21‐hydroxypregnenolone disulfate, with eight edges each (Figure S4a). All of their connected neighbors are steroid metabolites, implying central roles in cortisol homeostasis. Other nodes with high connectivity include N4‐acetylcytidine and 5,6‐dihydrouridine, with six edges each (Figure S4b). These were connected to each other by strongly correlated edges (Pearson correlationρ=0.87) in the third cluster. In the second cluster, defined by carnitine metabolism, carnitine and acetylcarnitine (C2) represented hubs with four connected edges (Figure S4c). Overall, the findings of network analysis revealed not only steroid biosynthesis, but other pathways also including fatty acid, pyrimidine, and carnitine metabolism, were altered due to adrenal suppression.

### 3.4 Validation in CAMP cohort

In total, 482 serum metabolites were analyzed for association with serum cortisol levels by linear regression; no metabolite was significantly associated with 24‐h urinary cortisol measurement using FDR adjusted *p‐value* threshold of 0.05. By unadjusted *p* < .05, 46 metabolites were potentially associated with urinary cortisol level (range of unadjusted *p‐values*: 6.12 × 10^−04^ to 4.99 × 10^−2^) (Table S4). Of these 46, four metabolites were also significantly associated with peak plasma cortisol measurement in the PASS cohort, matching with HMDB and KEGG IDs. These four were urocanic acid (trans‐urocanate), C2 carnitine (acetylcarnitine (C2)), uracil, and sorbitol (mannitol/sorbitol). For super and sub pathway annotation, 30 of 46 metabolites could be annotated to the lipid class with 21 metabolites that mainly belonged to fatty acid synthesis and metabolism. We also evaluated the association between serum metabolites with serum cortisol levels at different time points (baseline/30‐min/60‐min). However, no overlap of significant metabolites between PASS and CAMP were observed at the baseline cortisol level. Two overlapping and significant metabolites, kynurenine and pseudouridine, were identified at the 30‐min and 60‐ minute timepoints, respectively (Table S5).

## 4 DISCUSSION

Asthma patients exposed to high doses and long durations of ICS use are at increased risk of adrenal insufficiency due to the suppression of endogenous cortisol production. Adrenal suppression in asthmatics can occur even at standard corticosteroid doses, suggesting that the origination is multi‐factorial. However, clinical factors do not entirely account for this phenomenon. While moderate‐ and high‐dose ICS use is associated with adrenal suppression, frequent courses of oral corticosteroids, most commonly prescribed for asthma exacerbations, can also lead to HPA axis dysfunction.^39^ Contributing clinical factors include corticosteroid exposure (type, dose, formulation, pharmacokinetics, duration of treatment, route of administration), and disease status (asthma severity, presence of exacerbations, comorbidities).^40^ While biochemical factors promoting HPA axis insufficiency are likely to play a role, genetic factors, notably genetic variation, could also contribute.^17^, ^41^ A study of 62 asthmatic children on ICS determined an association of four HPA axis‐related single‐nucleotide polymorphism (SNP) with changes in serum cortisol levels before and after adrenocorticotropic hormone (ACTH) stimulation test.^41^ In a recent genome‐wide association study, Hawcutt et al. reported a significant association of a SNP in *PDGFD* with adrenal suppression occurring in children with asthma who were taking ICS.^17^ However, while genetic factors likely confer susceptibility to the development of adrenal suppression in asthmatics taking ICS, few studies have been conducted to investigate this.^19^, ^20^ To identify molecular drivers that regulate endogenous cortisol levels, we profiled 200 urine samples from a pediatric asthma patient cohort taking ICS. Of 571 fully annotated and non‐xenobiotic metabolites, 90 metabolites were significantly associated with adrenal status. Forty‐four of these 90 were also previously identified as significantly altered across multiple metabolomic studies of asthma patients.^18^, ^19^, ^38^, ^42^, ^43^, ^44^ Based upon the most recent findings from our research group, 21 metabolites identified in this study were also associated with ICS response in asthma across different cohorts.^18^, ^19^ Another recent study found five metabolites including isovalerylcarnitine (C5); cortolone glucuronide (1), cortolone glucuronide (2), xanthurenate, and taurochenodeoxycholic acid 3‐sulfate that were reduced by ICS in a dose dependent manner as compared with placebo.^20^ Significant enrichment of multiple pathways for metabolism of steroids, pyrimidines, fatty acids, carnitine, and sugar was observed in this study, highlighting that ICS can impact multiple pathways related to adrenal function in patients with asthma. These findings demonstrate the value for metabolomics profiling of asthma patients on ICS with a spectrum of adrenal function, providing unique metabolite signatures and insights into biological pathways contributing to the development of tertiary adrenal suppression among asthmatic children on ICS treatment.

Alterations in lipid profiles observed in our samples mainly involved changes in steroid and fatty acid metabolism. Lipids and steroids have demonstrated potent inflammatory effects in respiratory diseases. Network analysis revealed a primary cluster comprised of steroid metabolites while both the second and third clusters in the network analysis included fatty acid metabolites. Steroid metabolism can be affected by duration of ICS use and the changes in levels of steroid hormones were observed across multiple studies. This study identified 26 endogenous steroids and their derivatives that were significantly decreased among patients with low peak cortisol levels following ACTH stimulation test. Notably, these included DHEA‐S, a biomarker for adrenal suppression.^45^ A recent metabolomic study found 17 steroid metabolites from major steroid hormone biosynthesis sub pathways that were markedly decreased in asthma patients on ICS.^19^ While the originating biochemical events preceding both asthma and adrenal insufficiency are not known, these findings confirm ICS treatment in children could reduce production of steroid hormones in the body, leading to increased risk of adrenal suppression.

Metabolite network analysis identified a unique cluster defined by carnitine, and four fatty acyl carnitines involved in β‐oxidation (the most important pathway for fatty acid metabolism). All were significantly decreased in the adrenal insufficient patients. Carnitine has an important role in the transport of fatty acids into the mitochondrial matrix for β‐oxidation.^46^ Carnitine reduction has been reported during and after pediatric asthma exacerbations ^47^ and is associated with asthma severity.^48^ Furthermore, the four acylcarnitines (short chain (C2), 3‐hydroxyhexanoylcarnitine, (S)‐3‐hydroxybutyrylcarnitine, undecenoylcarnitine) were strongly correlated in the second cluster. Decreased levels of these acylcarnitines suggest an impaired fatty acid oxidation capacity in asthma patients with tertiary adrenal insufficiency. Multiple studies suggested that impaired β‐oxidation may play a role in the development or severity of asthma.^49^, ^50^, ^51^ Additionally, when β‐oxidation is defective, the alternative pathway, ω‐oxidation can occur, producing dicarboxylic acids from medium chain fatty acids.^52^ In our study, three dicarboxylate fatty acids including heptenedioate, 4‐octenedioate, and nonenedioate were significantly decreased in adrenal sufficient patients. These changes suggest that the ω‐oxidation pathway may be inhibited or impaired, indicating a disruption in the metabolism of long chain fatty acids. A prior metabolomics study performed by our group also demonstrated potential association between ω‐oxidation to exacerbation in asthma cases with ICS treatment.^18^

Histidine is an essential amino acid that plays an important role in various physiological process, including the synthesis of proteins, regulation of immune function, and neurotransmitter synthesis.^53^ The metabolism of histidine involves several enzymes and pathways that convert histidine into multiple metabolites including urocanic acid (UCA), which is reported to have a direct relationship with asthma; low levels of UCA were present in children with allergic asthma.^42^ Our study revealed an association of UCA including two isomers, cis and trans, with adrenal insufficiency. Both were significantly associated with adrenal suppression. Notably, UCA was also significantly linked with 24‐h urinary cortisol after HPA test in pediatric asthma patients in CAMP cohort. In our study, the concentration of both cis and trans‐UCA were significantly reduced in patients with adrenal insufficiency compared with adrenal sufficiency. Moreover, cis‐UCA was the key component in histidine metabolism which was significantly downregulated in adrenal insufficient samples based on pathway analysis, suggesting UCA can serve as an indicator of reduced histidine metabolism. Although cis‐urocanic acid has not been extensively studied in relation to asthma, some studies have suggested that cis‐urocanic acid may play a role in regulating immune function and inflammation in the skin.^54^, ^55^ Further studies are warranted to clarify the roles of UCA and its metabolites in asthma and ICS response.

Glucose metabolism involves several different biochemical pathways including glycolysis, gluconeogenesis, and the pentose phosphate pathway.^56^ In our study, mannitol/sorbitol was significantly increased in adrenal insufficient patients relative to those that were adrenal sufficient. This finding was also confirmed through validation in the CAMP cohort. Sorbitol was significantly negatively associated with 24‐h urinary cortisol level (estimated effect = −55.2 in linear model). Another study showed that mannitol/sorbitol was associated with increased frequency of asthma exacerbations while on ICS in adult patients from Mass General Brigham Biobank cohort.^18^ Sorbitol is converted to fructose, and under normal glucose homeostasis, conversion of less than 3% of glucose to sorbitol (polyol pathway) is the minor route of glucose metabolism, which is concurrent with glycolysis.^57^ Therefore, excess glucose could lead to a large formation of sorbitol, causing osmotic damage.^57^ Additionally, sorbitol dehydrogenase converts sorbitol to fructose, preventing sorbitol accumulation.^57^ However, in our study, only mannitol/sorbitol and glucose were significantly elevated in adrenal insufficient patients. Fructose levels were not significantly different between adrenal sufficient vs insufficient patients. This finding suggests that long‐term ICS use leading to adrenal insufficiency could induce altered glucose metabolism including hyperglycemia, and osmotic damage caused by increased levels of sorbitol. Further research is needed to determine the exact mechanism involved.

Another altered pathway with significantly elevated metabolites in adrenal insufficient patients was the γ‐glutamyl (GG) amino acid pathway. Based on ChemRICH analysis, **“**GG‐amino acid” was the only cluster containing all seven metabolites that were enriched in the adrenal insufficient patients. Of the seven, three GG‐amino acids (including GG‐tyrosine, GG‐epsilon‐lysine, and GG‐leucine) were significantly increased in the adrenal insufficient group based on logistic regression models. These metabolites play critical roles in maintaining glutathione homeostasis.^58^ In brief, the GG group is a small chemical moiety that is present in excess glutathione molecules that are produced by cells.^58^ When cells produce excess glutathione, the GG group can be transferred to amino acids or dipeptides by the enzyme GG, resulting in the formation of GG‐amino acid.^58^ Excess glutathione from cells is transferred to various amino acids or dipeptide acceptors to produce GG‐amino acids, mediated by the enzyme GG‐ transpeptidase (GGT).^58^ The levels of several GG‐amino acids showed significant increases in the adrenal insufficient patients in this study, suggesting elevated GGT enzyme activity indicating increased oxidative stress and inflammation.^59^ Furthermore, high GGT levels are connected to high levels of leukotriene and other inflammatory mediators, which can exacerbate asthma symptoms.^60^ In another epidemiological study, in healthy patients, a high serum GGT level (measured during general health checkup) was associated with the risk of future development of asthma, potentially through mechanisms related to increased oxidative stress.^61^

Recent studies showed that RNA modification is involved in multiple cellular processes, leading to alterations in immune responses.^62^ In this study, nine RNA modification molecules were significantly reduced in the adrenal insufficient patients. These include methylated nucleosides (N3‐methyluridine, 3‐methylcytidine), the RNA base uracil and its methylated derivative thymine, the RNA nucleoside pseudouridine, and the tRNA molecules 5,6‐dihydrouridine and 1‐methylguanine as well as a metabolite related to the highly conserved RNA modification process, N4‐acetycytidine. This finding suggests that the synthesis and degradation of various RNAs were decreased in adrenal insufficient patients. Seven of nine significant nucleotide molecules belonged to pyrimidine metabolism. Of these seven, uracil was significantly associated with cortisol levels in both CAMP and PASS cohorts. Uracil is involved in inflammation^63^ and levels were significantly decreased in severe asthma.^48^, ^64^ Pyrimidine metabolism was linked to asthma pathophysiology including methacholine responsiveness (pre‐ and postbronchodilator) in children.^23^ Another study in mice showed that several metabolites of pyrimidine metabolism including uracil were significantly decreased in asthma models and were significantly elevated after asthma treatment.^65^ However, there are few studies to date on the effects of altered RNA modification processes in asthma^66^, ^67^; further studies are needed to explore their potential relationship.

Our study reveals important changes in the urinary metabolome across multiple chemical classes in pediatric asthma patients taking ICS, providing evidence of alterations in multiple biochemical pathways in patients experiencing tertiary adrenal suppression. While our study has multiple strengths, we must mention several limitations. A major limitation of our study includes a low sample size for the adrenal insufficient group. The rate of adrenal suppression caused by long‐term corticosteroid use was higher in the PASS genetic study, which has a larger number of profiled samples relative to this subset of urine samples from the cohort. Not every individual provided urine samples, and adrenal status was blinded to investigators until analysis. Therefore, to account for potential bias between two groups, we performed SMOTE as a method to correct for imbalance. Additionally, while several sex steroid metabolites were significantly associated with adrenal status in our analysis, we could not interrogate the effect of gender related to the outcome due to the smaller sample size of the comparison group. Following methodological challenges, we identified additional limitations of the study, including lack of information on ICS dose included as a variable in our models; however, based on prior reports, the majority of children in this sample from PASS are taking high doses of ICS, which could be reflective of both asthma severity and susceptibility to adrenal suppression. Our study was also limited to inclusion of only white non‐Hispanic individuals and did not include individuals of other ethnic backgrounds, which is less informative for generalizing our findings. Furthermore, our results cannot be fully represented to all asthmatic patients, as we recruited only pediatric patients. Another limitation of this study is the small sample size of the CAMP validation cohort; metabolic profiling of the validation cohort did not share many metabolites with our discovery sing NetworkX. Proceedings of the 7th Python incohort, therefore we only validated four significant metabolites, although we were able to establish in silico relationships with a number of previously identified metabolites. As such, predictive modeling is currently not a robust approach with our current data set, due to the smaller sample size. With larger scale studies, we will have the capability to generate more robust predictive models to identify biomarkers of adrenal status in patients with asthma. From a clinical perspective, an important question that remains largely unanswered is whether (and how many) observed metabolite changes are related to underlying asthma versus solely tertiary adrenal suppression. There is likely a substantial molecular overlap between these phenotypes, but the extent of this, and its originating biological event(s), is not known. While our study design compared two groups based on adrenal status within the same cohort, there is likely to be confounding in regard to asthma severity and the presence of concomitant conditions. Given the limited power, we do not have sufficient numbers within these groups to enable stratified analyses that would help clarify this. However, we note that multiple metabolites associated with adrenal status were also previously associated with asthma and ICS use, suggesting that at least some of the observed changes are related to asthma progression and ICS exposure.

In conclusion, our findings demonstrate that there are significant differences in urinary metabolite profiles between adrenal sufficient versus insufficient pediatric asthma patients who are taking ICS. The study highlighted potential metabolomic indicators of adrenal suppression occurring while on ICS. Notably, several anti‐inflammatory metabolites were decreased in adrenal insufficient patients, underlying the HPA‐axis impact of ICS use via immune and inflammatory responses. These metabolites may serve as biomarkers of both ICS response and risk of adrenal suppression in asthma patients. Ongoing studies to integrate these findings with other omics data will ultimately deepen our understanding of the impact of ICS exposure on adrenocortical function in asthma patients.

## AUTHOR CONTRIBUTIONS

Amber Dahlin, Scott T. Weiss, Jessica Lasky‐Su, and Munir Pirmohamed conceptualized the study. All authors contributed to the writing and revised the initial manuscript. Amber Dahlin, Jessica Lasky‐Su, Scott T. Weiss supervised the statistical analysis and interpreted the data. Amber Dahlin obtained funding for this research. Munir Pirmohamed recruited patients for the PASS cohort. Dung T. Tran carried out the main statistical analysis, drafted the initial manuscript. All authors approved the submitted version.

## CONFLICT OF INTEREST STATEMENT

The authors declare no conflict of interest.

## ETHICS STATEMENT

The project was reviewed and approved by the Brigham and Women's Hospital Institutional Review Board (IRB: protocol# 2020P000591) with a waiver of informed consent.

## Supporting information

Supporting information.

Supporting information.

Supporting information.

Supporting information.

Supporting information.

Supporting information.

## Data Availability

The metabolomics and metadata reported in this paper will be made available by request following publication.
