# Supplementary material for: Urine metabolomics signature reveals novel determinants of adrenal suppression in children taking inhaled corticosteroids to control asthma symptoms
Source: Immun Inflamm Dis. 2024 Jul 19;12(7):e1315. doi: 10.1002/iid3.1315 (PMC11259003; doi:10.1002/iid3.1315)
Supplement: Supplementary file 1 — Supporting information. [file IID3-12-e1315-s005.docx]

**Title**

Urine metabolomics signature reveals novel determinants of adrenal suppression in children taking inhaled corticosteroids to control asthma symptoms

**Authors**

Dung Tran, MS, PhD^1^, Yulu Chen PhD^1^, Yi Zheng PhD, MPH^1^, Julian Hecker PhD^1^, Daniel B Hawcutt MD^2^, Munir Pirmohamed MD^2^, Jessica Lasky-Su, M.S., ScD^1^, Ann C. Wu, MD^1,3^, Kelan G. Tantisira, MD, MPH^4^, Michael J. McGeachie PhD^1^, Scott T. Weiss, MD, MS^1^, and Amber Dahlin, PhD, MMSc^1^.

**Author Affiliations**

^1^Channing Division of Network Medicine, Brigham and Women’s Hospital and Harvard Medical School, Boston, MA USA

^2^University of Liverpool, Liverpool, UK

^3^Department of Population Medicine, Harvard Medical School and Harvard Pilgrim Health Care Institute, Boston, MA USA

^4^Division of Pediatric Respiratory Medicine, University of California San Diego and Rady Children’s Hospital, San Diego, CA USA

**Comparing SMOTE based models and others**

Synthetic Minority Oversampling Technique (SMOTE) ^1^ is a data synthetizing method that is used to oversample data in the minor group in a unbalanced distribution. In this sub-study, we conduct a simulation to evaluate the quality of metabolites detected by SMOTE compared with regular Logistics regression as well as SPA models. Saddle point Approximation (SPA) is a special logistic Regression model that uses saddle point approximation instead of the traditional approximation method to estimate the models’ coefficients ^2^. More specifically, the traditional optimization method for logistic regression approximates the null distribution using a normal distribution that depends only on the mean and standard deviation of the score statistics. On the other hand, saddle point approximation is an improvement over the traditional method in that it uses the entire cumulant-generating function. Overall, the SPA model can handle unbalanced data better than regular Logistic regression. In terms of experiment, we set up the simulation to match our data settings, i.e., a dataset of 200 samples with 188 belonging to one group and 12 the other. Another assumption is that data small size of the minor group may not be enough to represent its original population. Therefore, we start from a dataset of 188 samples each group, then keep 188 from one group, and randomly pick only 12 from the other, to form the final dataset of 200 samples. Before the sampling of minor group, we simulate a predictor which associates to the group at random levels. A logistic regression is fitted on the unsampled data to determine the true significance of the predictor. Then, in the sampled data, we performed SMOTE, Borderline SMOTE, unweighted logistic regression, and SPA, and recorded each method’s response on whether the predictor is significant. The experiment is repeated for 10,000 runs at three true significance levels, 95%, 55%, and 5%. Finally, we calculated the models’ precision and recall at the three significance levels which are illustrated in Supplementary Figure S5. Our simulations found that in the unbalanced data, SMOTE-based models tended to discover a larger number of significant true positive metabolites, however, with a higher rate of false positives (Supplementary Figure S5). SPA, on the other hand, was able to maintain low rates of false positives, albeit with the trade-off of a very low number of true positives and a high number of false negatives (Supplementary Figure S5). Therefore, we decided to use SMOTE as the preferred method for this analysis. To reduce false positives, we combined multiple approaches including logistic regression, and OPLS-DA, and performed an independent validation of top metabolites using a related dataset and current findings from the literature.

**References**

1. Chawla N, Bowyer K, Hall L, Kegelmeyer W. SMOTE: Synthetic Minority Over-sampling Technique. *J Artif Intell Res (JAIR)* 2002; **16**: 321-57.

2. Ma C, Blackwell T, Boehnke M, Scott LJ. Recommended joint and meta-analysis strategies for case-control association testing of single low-count variants. *Genet Epidemiol* 2013; **37**(6): 539-50.

**Supplementary Figures**

**
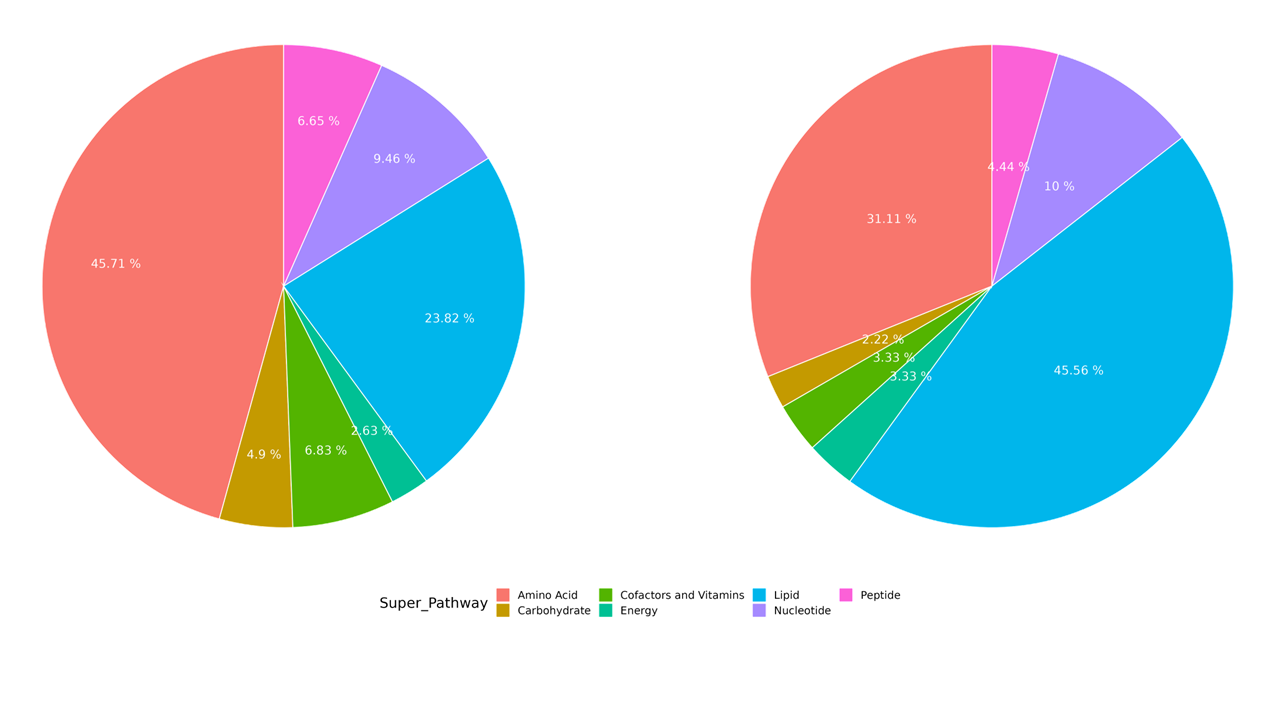
**

Supplementary Figure 1: Super pathway distribution of (a) 571 metabolites after quality control and (b) 90 significant metabolites from logistic regression models.

**
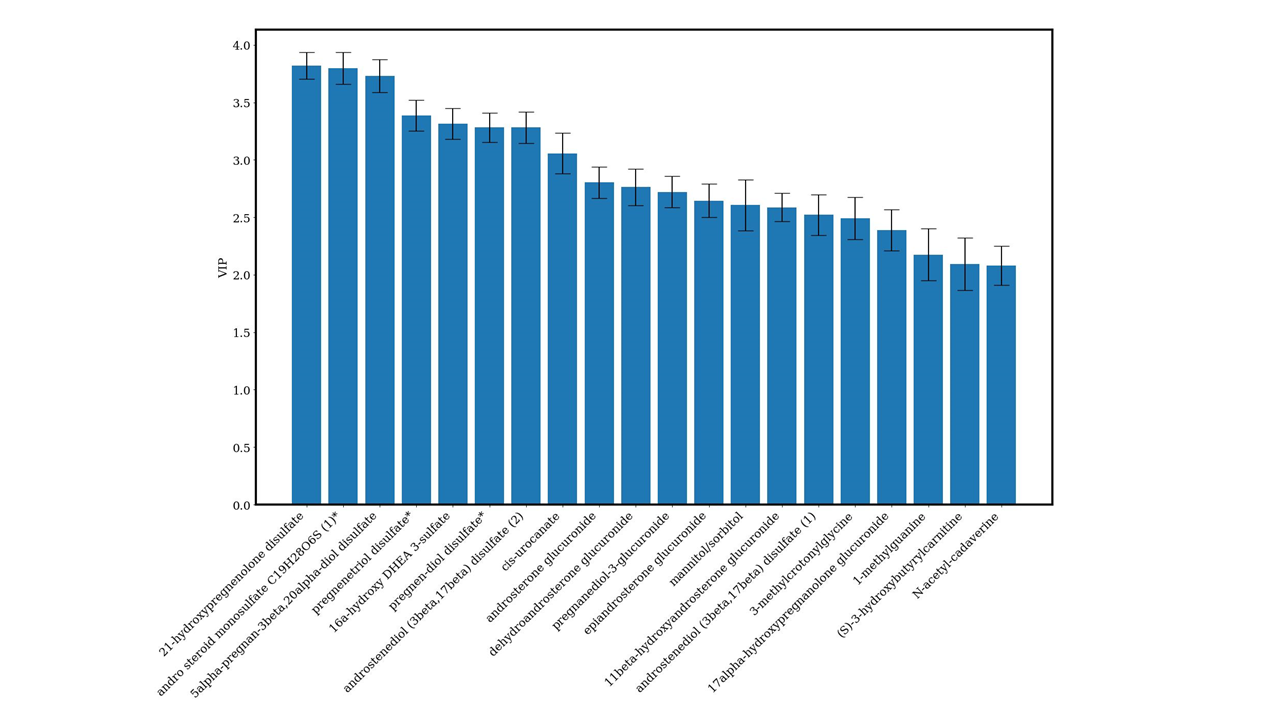
**

Supplementary Figure 2: VIP score plot derived from the OPLS-DA analysis highlighting the top 20 metabolites of discriminatory significance.

**
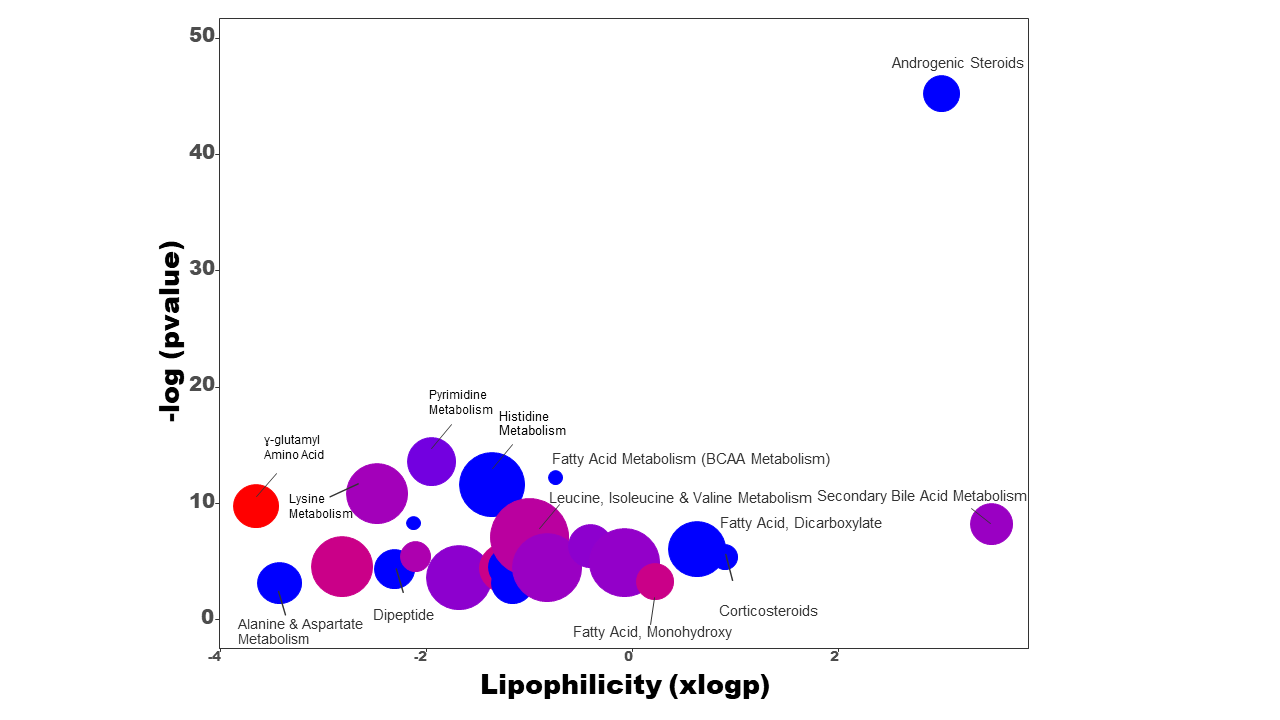
**

Supplementary Figure 3: ChemRICH set enrichment statistics plot. Each node reflects a significantly altered cluster of metabolites related to adrenal suppression. Enrichment *p-values* are given by the Kolmogorov-Smirnov-test. Node size represents the total number of metabolites in each cluster set. The node color scale shows the proportion of increased (red) or decreased (blue) compounds in adrenal insufficient compared to adrenal sufficient patients. Purple nodes include both increased and decreased metabolites.

**
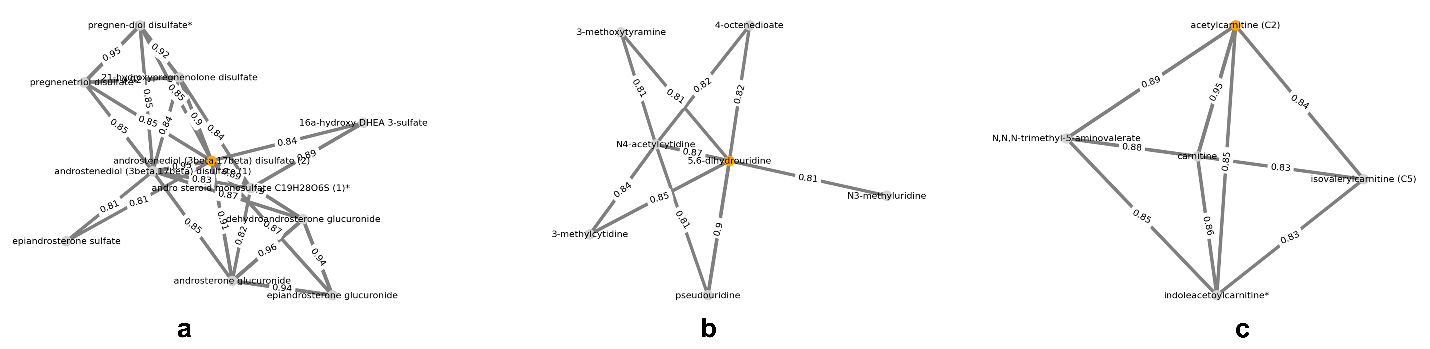
**

Supplementary Figure 4: Metabolite neighborhood of a) androstenediol (3beta,17beta) disulfate (2); b) 5,6-dihydrouridine; c) acetylcarnitine (C2).

**
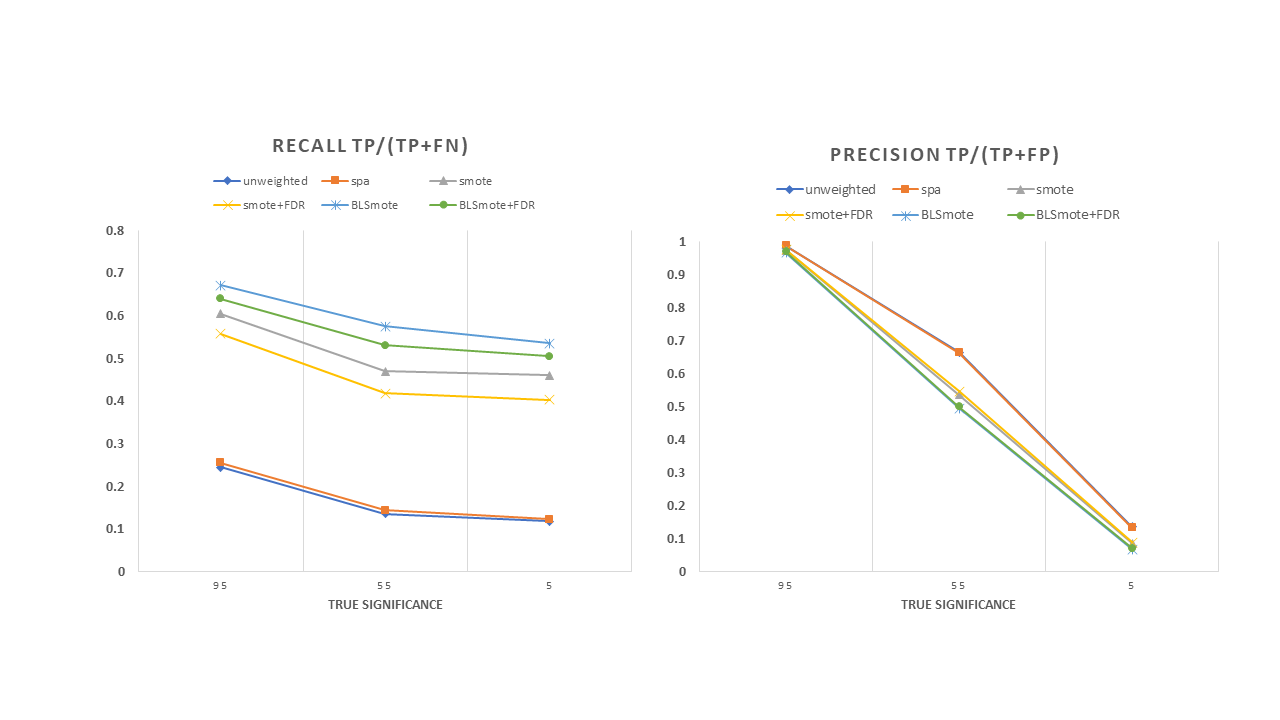
**Supplementary Figure 5: Precision and recall of models in simulation.
