# Supplementary material for: Urine metabolomics signature reveals novel determinants of adrenal suppression in children taking inhaled corticosteroids to control asthma symptoms
Source: Immun Inflamm Dis. 2024 Jul 19;12(7):e1315. doi: 10.1002/iid3.1315 (PMC11259003; doi:10.1002/iid3.1315)
Supplement: Supplementary file 2 — Supporting information. [file IID3-12-e1315-s002.pdf]

Supplementary Table 1 Urinary metabolites that are significantly different between patients with adrenal sufficient vs insufficient (FDR adjusted p-value < 0.05). Metabolites are grouped by metabolic sub-pathway and in order of decreasing significance.

No	Metabolites	Super Pathway	Sub Pathway	KEGG	HMDB	PubChem	SMILES	OR (95% CI)	FDR p value	VIP Score	In Silicon Replication	Citation
1	N,N-dimethylalanine	Amino Acid	Alanine and Aspartate Metabolism	NA	NA	5488191	C[C@@H](C(=O)O)N(C)C	0.49 (0.33,0.73)	7.85E-03	1.51	NA	NA
2	andro steroid monosulfate C19H28O6S (1)*	Lipid	Androgenic Steroids	C04555	HMDB02759	NA	NA	0.3 (0.21,0.43)	1.21E-08	3.8	Steroid metabolite associations between asthma cases and controls; asthma cases with ICS intake and controls; and asthma cases with and without ICS intake in MGBB-Asthma	1
3	androstenediol (3beta,17beta) disulfate (2)	Lipid	Androgenic Steroids	C04295	HMDB03818	87120982	C[C@]12CC[C@H]3[C@H]([C@@H]1CC[C@@H]2OS(=O)(=O)O)CCC4=C[C@H](CC[C@]34C)OS(=O)(=O)O	0.32 (0.22,0.46)	1.14E-07	3.28	Steroid metabolite associations between asthma cases and controls; asthma cases with ICS intake and controls; asthma cases with no ICS and controls; and asthma cases with and without ICS intake in MGBB-Asthma	1
4	16a-hydroxy DHEA 3-sulfate	Lipid	Androgenic Steroids	NA	HMDB0062544	20848951	C[C@]12CC[C@@H](CC1=CC[C@@H]3[C@@H]2CC[C@]4([C@H]3C[C@H](C4=O)O)C)OS(=O)(=O)O	0.39 (0.29,0.53)	1.31E-07	3.31	Plasma metabolites significantly associated with asthma in EPIC-Norfolk with replication in MGBB Asthma	1
5	androsterone glucuronide	Lipid	Androgenic Steroids	C11135	HMDB02829	114833	C[C@]12CC[C@H](C[C@@H]1CC[C@@H]3[C@@H]2CC[C@]4([C@H]3CCC4=O)C)O[C@H]5[C@@H]([C@H]([C@@H]([C@H](O5)C(=O)O)O)O)O	0.41 (0.29,0.57)	9.60E-06	2.8	Steroid metabolite associations between asthma cases and controls; asthma cases with ICS intake and controls in MGBB-Asthma	1
6	dehydroandrosterone glucuronide	Lipid	Androgenic Steroids	NA	NA	NA	NA	0.43 (0.31,0.59)	1.18E-05	2.76	NA	NA

No	Metabolites	Super Pathway	Sub Pathway	KEGG	HMDB	PubChem	SMILES	OR (95% CI)	FDR p value	VIP Score	In Silicon Replication	Citation
7	androstenediol (3beta,17beta) disulfate (1)	Lipid	Androgenic Steroids	C04295	HMDB03818	87120982	C[C@]12CC[C@H]3[C@H]([C@@H]1CC[C@@H]2OS(=O)(=O)O)CCC4=C[C@H](CC[C@]34C)OS(=O)(=O)O	0.46 (0.34,0.62)	2.26E-05	2.52	Steroid metabolite associations between asthma cases and controls; asthma cases with ICS intake and controls; and asthma cases with and without ICS intake in MGBB-Asthma	1
8	epiandrosterone glucuronide	Lipid	Androgenic Steroids	NA	NA	10298641	C[C@]12CC[C@@H](C[C@@H]1CC[C@@H]3[C@@H]2CC[C@]4([C@H]3CCC4=O)C)O[C@H]5[C@@H]([C@H]([C@@H]([C@H](O5)C(=O)O)O)O)O	0.47 (0.35,0.64)	4.18E-05	2.65	NA	NA
9	11beta-hydroxyandrosterone glucuronide	Lipid	Androgenic Steroids	NA	NA	NA	NA	0.37 (0.25,0.56)	7.38E-05	2.59	Plasma metabolites significantly associated with asthma in EPIC-Norfolk with replication in MGBB Asthma	1
10	dehydroepiandrosterone sulfate (DHEA-S)	Lipid	Androgenic Steroids	C04555	HMDB01032	12594	C[C@]12CC[C@H]3[C@H]([C@@H]1CCC2=O)CC=C4[C@@]3(CC[C@@H](C4)OS(=O)(=O)O)C	0.53 (0.4,0.69)	1.42E-04	2.08	Plasma metabolites significantly associated with asthma in EPIC-Norfolk with replication in MGBB Asthma	1
11	epiandrosterone sulfate	Lipid	Androgenic Steroids	NA	HMDB0062657	9929317	C[C@]12CC[C@@H](C[C@@H]1CC[C@@H]3[C@@H]2CC[C@]4([C@H]3CCC4=O)C)OS(=O)(=O)O	0.57 (0.43,0.74)	1.01E-03	1.86	Plasma metabolites significantly associated with asthma in EPIC-Norfolk with replication in MGBB Asthma	1
12	11beta-hydroxyandrosterone sulfate (2)	Lipid	Androgenic Steroids	NA	NA	NA	NA	0.6 (0.45,0.8)	7.88E-03	1.84	NA	NA

No	Metabolites	Super Pathway	Sub Pathway	KEGG	HMDB	PubChem	SMILES	OR (95% CI)	FDR p value	VIP Score	In Silicon Replication	Citation
13	etiocholanolone glucuronide	Lipid	Androgenic Steroids	C11136	HMDB04484	443078	C[C@]12CC[C@H](C[C@H]1CC[C@@H]3[C@@H]2CC[C@]4([C@H]3CCC4=O)C)O[C@H]5[C@@H]([C@H]([C@@H]([C@H](O5)C(=O)O)O)O)O	0.68 (0.53,0.86)	1.57E-02	1.54	Plasma metabolites significantly associated with asthma in EPIC-Norfolk with replication in MGBB Asthma	1
14	11-ketoetiocholanolone sulfate	Lipid	Androgenic Steroids	NA	NA	NA	NA	0.65 (0.48,0.87)	3.25E-02	1.62	NA	NA
15	ascorbic acid 3-sulfate*	Cofactors and Vitamins	Ascorbate and Aldarate Metabolism	NA	HMDB0240641	11425365	C([C@@H]([C@@H]1C(=C(C(=O)O1)O)OS(=O)(=O)O)O)O	1.58 (1.16,2.16)	2.95E-02	1.46	NA	NA
16	carnitine	Lipid	Carnitine Metabolism	C00318	HMDB00062	10917	C[N+](C)(C)C[C@@H](CC(=O)[O-])O	0.61 (0.44,0.84)	2.20E-02	1.37	Altered carnitine metabolism is independent of OCS and associated with mitochondrial dysfunction, presenting a potential target for intervention	9
17	3alpha,21-dihydroxy-5beta-pregnane-11,20-dione 21-glucuronide	Lipid	Corticosteroids	NA	NA	NA	NA	0.56 (0.42,0.75)	1.98E-03	1.97	NA	NA
18	11-dehydrocorticosterone sulfate	Lipid	Corticosteroids	NA	NA	NA	NA	0.57 (0.43,0.76)	2.10E-03	1.86	NA	NA
19	cortisol 21-sulfate	Lipid	Corticosteroids	C02822	HMDB62779	102172	C[C@]12CCC(=O)C=C1CC[C@@H]3[C@@H]2[C@H](C[C@]4([C@H]3CC[C@@]4(C(=O)COS(=O)(=O)O)O)C)O	0.62 (0.47,0.82)	1.16E-02	1.65	NA	NA
20	cortolone glucuronide (1)	Lipid	Corticosteroids	NA	NA	NA	NA	0.61 (0.44,0.84)	2.32E-02	1.58	Significantly reduced by inhaled GCs in a dose-dependent manner compared with placebo	13
21	cortolone glucuronide (2)	Lipid	Corticosteroids	NA	NA	NA	NA	0.62 (0.46,0.85)	2.40E-02	1.6	Significantly reduced by inhaled GCs in a dose-dependent manner compared with placebo	13

No	Metabolites	Super Pathway	Sub Pathway	KEGG	HMDB	PubChem	SMILES	OR (95% CI)	FDR p value	VIP Score	In Silicon Replication	Citation
22	tetrahydrocortisol	Lipid	Corticosteroids	C05472	HMDB00949	44725717	C[C@]12CC[C@H](CC1CCC3C2[C@H](C[C@]4(C3CC[C@@]4(C(=O)CO)O)C)O)O	0.63 (0.46,0.86)	2.89E-02	1.4	Plasma metabolites significantly associated with prevalent asthma outcome in discovery EPIC-Norfolk cohort	1
23	cortisone	Lipid	Corticosteroids	C00762	HMDB02802	222786	C[C@]12CCC(=O)C=C1CC[C@@H]3[C@@H]2C(=O)C[C@]4([C@H]3CC[C@@]4(C(=O)CO)O)C	0.65 (0.47,0.89)	5.00E-02	1.35	Metabolite (predictor) associations with exacerbation (outcome) in asthma cases with inhaled corticosteroid (ICS) intake (negative assocation)	4
24	guanidinoacetate	Amino Acid	Creatine Metabolism	C00581	HMDB00128	763	C(C(=O)O)N=C(N)N	0.54 (0.36,0.81)	2.42E-02	1.69	NA	NA
25	phenylalanylglycine	Peptide	Dipeptide	NA	HMDB28995	98207	C1=CC=C(C=C1)CC(C(=O)NCC(=O)O)N	0.56 (0.37,0.83)	3.40E-02	1.46	NA	NA
26	pimeloylcarnitine/3-methyladipoylcarnitine (C7-DC)	Lipid	Fatty Acid Metabolism (Acyl Carnitine, Dicarboxylate)	NA	NA	NA	NA	0.48 (0.32,0.73)	8.80E-03	1.5	Metabolites significantly increased with increased Procalcitonin over days 0–7. Procalcitonin is a biomarker of systemic inflammation and may have importance in the immune response	10
27	(S)-3-hydroxybutyrylcarnitine	Lipid	Fatty Acid Metabolism (Acyl Carnitine, Hydroxy)	NA	HMDB13127	155907023	CC(CC(=O)[C@](CC(=O)[O-])(C[N+](C)(C)C)O)O	0.47 (0.33,0.66)	4.85E-04	2.09	NA	NA
28	3-hydroxyhexanoylcarnitine (1)	Lipid	Fatty Acid Metabolism (Acyl Carnitine, Hydroxy)	NA	NA	NA	NA	0.6 (0.42,0.86)	3.41E-02	1.33	NA	NA
29	undecenoylcarnitine (C11:1)	Lipid	Fatty Acid Metabolism (Acyl Carnitine, Monounsaturated)	NA	NA	NA	NA	0.6 (0.45,0.79)	4.51E-03	1.68	NA	NA

No	Metabolites	Super Pathway	Sub Pathway	KEGG	HMDB	PubChem	SMILES	OR (95% CI)	FDR p value	VIP Score	In Silicon Replication	Citation
30	acetylcarnitine (C2)	Lipid	Fatty Acid Metabolism (Acyl Carnitine, Short Chain)	C02571	HMDB00201	1	CC(=O)OC(CC(=O)[O-])C[N+](C)(C)C	0.6 (0.44,0.81)	9.79E-03	1.56	carnitines, which decreased linearly with disease severity in the non-smoking groups	9
31	4-methylhexanoylglutamine	Lipid	Fatty Acid Metabolism (Acyl Glutamine)	NA	NA	NA	NA	0.62 (0.44,0.86)	3.32E-02	1.5	NA	NA
32	trans-2-hexenoylglycine	Lipid	Fatty Acid Metabolism (Acyl Glycine)	NA	NA	NA	NA	0.6 (0.43,0.83)	2.24E-02	1.65	NA	NA
33	2-aminoheptanoate	Lipid	Fatty Acid, Amino	NA	NA	227939	CCCCCC(C(=O)O)N	0.66 (0.49,0.9)	4.86E-02	1.33	NA	NA
34	nonenedioate (C9:1-DC)*	Lipid	Fatty Acid, Dicarboxylate	NA	NA	NA	NA	0.49 (0.33,0.71)	3.69E-03	1.83	NA	NA
35	heptenedioate (C7:1-DC)*	Lipid	Fatty Acid, Dicarboxylate	NA	NA	NA	NA	0.47 (0.31,0.71)	4.69E-03	1.73	NA	NA
36	4-octenedioate	Lipid	Fatty Acid, Dicarboxylate	NA	HMDB04982	11805205	C(CC(=O)O)/C=C\CCC(=O)O	0.53 (0.35,0.79)	1.72E-02	1.39	NA	NA
37	mannitol/sorbitol	Carbohydrate	Fructose, Mannose and Galactose Metabolism	C00794	HMDB00247	5780	C([C@H]([C@H]([C@@H]([C@H](CO)O)O)O)O)O	2.94 (1.9,4.55)	4.97E-05	2.61	Metabolite (predictor) associations with exacerbation (outcome) in asthma cases with inhaled corticosteroid (ICS) intake (possitive assocation)	4
38	gamma-glutamyltyrosine	Peptide	Gamma-glutamyl Amino Acid	NA	HMDB11741	94340	C1=CC(=CC=C1C[C@@H](C(=O)O)NC(=O)CC[C@@H](C(=O)O)N)O	2.25 (1.53,3.31)	8.87E-04	2.04	Metabolites with significant fold change between asthma and healthy control subjects	6
39	gamma-glutamyl-epsilon-lysine	Peptide	Gamma-glutamyl Amino Acid	NA	HMDB03869	7015685	C(CCNC(=O)CC[C@@H](C(=O)O)N)C[C@@H](C(=O)O)N	1.9 (1.26,2.88)	2.22E-02	1.56	NA	NA
40	gamma-glutamylleucine	Peptide	Gamma-glutamyl Amino Acid	NA	HMDB11171	151023	CC(C)C[C@@H](C(=O)O)NC(=O)CC[C@@H](C(=O)O)N	1.77 (1.19,2.62)	3.42E-02	1.49	NA	NA
41	2-hydroxybutyrate/2-hydroxyisobutyrate	Amino Acid	Glutathione Metabolism	NA	NA	NA	NA	2.11 (1.26,3.54)	3.46E-02	1.43	Two other butyrates, 2-hydroxyisobutyric acid and 2-hydroxybutyrate, have been implicated in children with asthma or AD	15

No	Metabolites	Super Pathway	Sub Pathway	KEGG	HMDB	PubChem	SMILES	OR (95% CI)	FDR p value	VIP Score	In Silicon Replication	Citation
42	glucose	Carbohydrate	Glycolysis, Gluconeogenesis, and Pyruvate Metabolism	C00031	HMDB00122	79025	C([C@@H]1[C@H]([C@@H]([C@H]([C@H](O1)O)O)O)O)O	2 (1.35,2.95)	7.02E-03	1.85	Fasting and 2-h blood glucose values were significantly higher in patients with asthma bronchiale than in control subjects	8
43	cis-urocanate	Amino Acid	Histidine Metabolism	NA	HMDB34174	5461073	C1=C(NC=N1)/C=C\C(=O)[O-]	0.2 (0.12,0.35)	1.42E-06	3.06	Urocanic acid related to inflammation/immunity and Ile-Pro to prolidase activity. Low levels in children with atopic asthma	2
44	formiminoglutamate	Amino Acid	Histidine Metabolism	C00439	HMDB00854	439233	C(CC(=O)O)[C@@H](C(=O)O)N=CN	0.53 (0.36,0.77)	1.01E-02	1.7	Formiminoglutamate (FIGLU) is a functional marker of insufficiency of folic acid, another B-vitamin. And there might be a casual link between maternal folic acid supplementary and the development of asthma in babies	11
45	trans-urocanate	Amino Acid	Histidine Metabolism	C00785	HMDB00301	736715	C1=C(NC=N1)/C=C/C(=O)O	0.52 (0.35,0.77)	1.29E-02	1.91	Biochemicals significantly different between FENO phenotype (exhaled nitric oxide)	6
46	anserine	Amino Acid	Histidine Metabolism	C01262	HMDB00194	112072	CN1C=NC=C1C[C@@H](C(=O)O)NC(=O)CCN	0.7 (0.55,0.9)	3.59E-02	1.19	NA	NA
47	homocarnosine	Amino Acid	Histidine Metabolism	C00884	HMDB00745	10243361	C1=C(NC=N1)C[C@@H](C(=O)O)NC(=O)CCCN	0.59 (0.4,0.87)	4.61E-02	1.24	NA	NA
48	myo-inositol	Lipid	Inositol Metabolism	C00137	HMDB00211	892	C1(C(C(C(C(C1O)O)O)O)O)O	1.76 (1.24,2.5)	1.64E-02	1.62	Myo-Ins is utilized in the field of pulmonology to treat respiratory conditions such as asthma, chronic obstructive pulmonary disease, and respiratory tract infections	12
49	3-methylcrotonylglycine	Amino Acid	Leucine, Isoleucine and Valine Metabolism	C20828	HMDB00459	169485	CC(=CC(=O)NCC(=O)O)C	3.07 (1.86,5.08)	3.34E-04	2.49	Elevated 3-methylcrotonylglycine in a asthma patient	5

No	Metabolites	Super Pathway	Sub Pathway	KEGG	HMDB	PubChem	SMILES	OR (95% CI)	FDR p value	VIP Score	In Silicon Replication	Citation
50	3-methylglutarylcarnitine (2)	Amino Acid	Leucine, Isoleucine and Valine Metabolism	NA	HMDB00552	128145	CC(CC(=O)[O-])CC(=O)OC(CC(=O)O)C[N+](C)(C)C	0.44 (0.29,0.67)	2.40E-03	1.82	NA	NA
51	tigloylglycine	Amino Acid	Leucine, Isoleucine and Valine Metabolism	NA	HMDB00959	6441567	C/C=C(\C)/C(=O)NCC(=O)O	2.57 (1.54,4.28)	4.98E-03	1.97	Higher levels in early-onset asthma compared with transient wheezers	7
52	2,3-dimethylsuccinate	Amino Acid	Leucine, Isoleucine and Valine Metabolism	NA	NA	11848	CC(C(C)C(=O)O)C(=O)O	2.06 (1.39,3.05)	5.42E-03	1.78	NA	NA
53	isovalerylcarnitine (C5)	Amino Acid	Leucine, Isoleucine and Valine Metabolism	NA	HMDB00688	169235	CC(C)CC(=O)O[C@H](CC(=O)[O-])C[N+](C)(C)C	0.62 (0.46,0.84)	1.92E-02	1.29	Significantly increased at all Fluticasone furoate doses	13
54	methylsuccinoylcarnitine	Amino Acid	Leucine, Isoleucine and Valine Metabolism	NA	NA	NA	NA	0.55 (0.36,0.85)	4.65E-02	1.16	NA	NA
55	N-acetyl-cadaverine	Amino Acid	Lysine Metabolism	NA	HMDB02284	189087	CC(=O)NCCCCCN	0.37 (0.24,0.59)	5.03E-04	2.08	NA	NA
56	N,N,N-trimethyl-5-aminovalerate	Amino Acid	Lysine Metabolism	NA	HMDB0240732	14274897	C[N+](C)(C)CCCCC(=O)[O-]	0.54 (0.39,0.76)	5.03E-03	1.62	NA	NA
57	taurine	Amino Acid	Methionine, Cysteine, SAM and Taurine Metabolism	C00245	HMDB00251	1123	C(CS(=O)(=O)O)N	0.58 (0.41,0.81)	1.56E-02	1.56	Higher level in the bronchoalveolar lavage (BAL) fluid of individuals with asthma	6
58	nicotinamide N-oxide	Cofactors and Vitamins	Nicotinate and Nicotinamide Metabolism	NA	HMDB02730	72661	C1=CC(=C[N+](=C1)[O-])C(=O)N	0.68 (0.52,0.89)	4.06E-02	1.26	NA	NA
59	phosphate	Energy	Oxidative Phosphorylation	C00009	HMDB01429	1004	OP(=O)(O)O	0.59 (0.46,0.75)	5.17E-04	1.99	NA	NA
60	5-methylthioadenosine (MTA)	Amino Acid	Polyamine Metabolism	C00170	HMDB01173	439176	CSC[C@@H]1[C@H]([C@H]([C@@H](O1)N2C=NC3=C(N=CN=C32)N)O)O	1.89 (1.27,2.8)	1.77E-02	1.55	Metabolite (predictor) associations with exacerbation (outcome) in asthma cases with inhaled corticosteroid (ICS) intake (possitive assocation)	4

No	Metabolites	Super Pathway	Sub Pathway	KEGG	HMDB	PubChem	SMILES	OR (95% CI)	FDR p value	VIP Score	In Silicon Replication	Citation
61	21-hydroxypregnenolone disulfate	Lipid	Pregnenolone Steroids	NA	NA	644042	CC(=O)[C@]1(CC[C@@H]2[C@@]1(CC[C@H]3[C@H]2CC=C4[C@@]3(CC[C@@H](C4)OS(=O)(=O)O)C)C)O	0.22 (0.14,0.34)	1.18E-08	3.82	Plasma metabolites significantly associated with asthma in EPIC-Norfolk with replication in MGBB Asthma	1
62	pregnen-diol disulfate*	Lipid	Pregnenolone Steroids	NA	NA	NA	NA	0.28 (0.19,0.41)	3.22E-08	3.28	Plasma metabolites significantly associated with prevalent asthma outcome in discovery EPIC-Norfolk cohort	1
63	pregnenetriol disulfate*	Lipid	Pregnenolone Steroids	NA	NA	NA	NA	0.29 (0.2,0.43)	7.32E-08	3.38	Plasma metabolites significantly associated with asthma in EPIC-Norfolk with replication in MGBB Asthma	1
64	17alpha-hydroxypregnanolone glucuronide	Lipid	Pregnenolone Steroids	NA	NA	NA	NA	0.53 (0.41,0.68)	3.43E-05	2.39	Maternal baseline (10–18 weeks gestation) metabolites significantly associated with child asthma/recurrent wheeze status by age 3 in girls in VDAART	3
65	5alpha-pregnan-3beta,20alpha-diol disulfate	Lipid	Progestin Steroids	NA	HMDB0094650	5748360	C[C@@H]([C@H]1CC[C@@H]2[C@@]1(CC[C@H]3[C@H]2CC[C@@H]4[C@@]3(CC[C@@H](C4)OS(=O)(=O)O)C)C)OS(=O)(=O)O	0.25 (0.16,0.37)	1.10E-08	3.73	Plasma metabolites significantly associated with prevalent asthma outcome in discovery EPIC-Norfolk cohort	1
66	pregnanediol-3-glucuronide	Lipid	Progestin Steroids	C03033	HMDB10318	123796	C[C@@H]([C@H]1CC[C@@H]2[C@@]1(CC[C@H]3[C@H]2CC[C@H]4[C@@]3(CC[C@H](C4)O[C@H]5[C@@H]([C@H]([C@@H]([C@H](O5)C(=O)O)O)O)O)C)C)O	0.44 (0.32,0.61)	2.69E-05	2.72	Steroid metabolite associations between asthma cases and controls in MGBB-Asthma	1

No	Metabolites	Super Pathway	Sub Pathway	KEGG	HMDB	PubChem	SMILES	OR (95% CI)	FDR p value	VIP Score	In Silicon Replication	Citation
67	1-methylhypoxanthine	Nucleotide	Purine Metabolism, (Hypo)Xanthine/Inosine containing	NA	HMDB13141	70765	CN1C=NC2=C(C1=O)NC=N2	0.42 (0.24,0.73)	1.94E-02	1.66	NA	NA
68	1-methylguanine	Nucleotide	Purine Metabolism, Guanine containing	C04152	HMDB03282	70315	CN1C(=O)C2=C(N=CN2)N=C1N	0.48 (0.33,0.69)	1.50E-03	2.18	NA	NA
69	3-methylcytidine	Nucleotide	Pyrimidine Metabolism, Cytidine containing	NA	NA	159649	CN1C(=N)C=CN(C1=O)[C@@H]2[C@@H]([C@@H]([C@H](O2)CO)O)O	0.5 (0.31,0.81)	3.64E-02	1.33	Significantly associated with asthma	17
70	N4-acetylcytidine	Nucleotide	Pyrimidine Metabolism, Cytidine containing	C22293	HMDB05923	107461	CC(=O)NC1=NC(=O)N(C=C1)[C@H]2[C@@H]([C@@H]([C@H](O2)CO)O)O	0.54 (0.35,0.84)	4.10E-02	1.3	The recent progress in N4-Acetylcytidine on RNA expression is also playing a key role on the development of asthma	19
71	thymine	Nucleotide	Pyrimidine Metabolism, Thymine containing	C00178	HMDB00262	1135	CC1=CNC(=O)NC1=O	0.57 (0.38,0.86)	4.55E-02	1.16	NA	NA
72	uracil	Nucleotide	Pyrimidine Metabolism, Uracil containing	C00106	HMDB00300	1174	C1=CNC(=O)NC1=O	0.48 (0.32,0.73)	7.87E-03	1.61	Metabolites that most strongly drove this separation included short-chain acylcarnitines, histidine, taurine, uracil, 2-deoxyinosine, kynurenic acid (decreased in severe asthma)	9
73	5,6-dihydrouridine	Nucleotide	Pyrimidine Metabolism, Uracil containing	NA	HMDB0000497	94312	C1CN(C(=O)NC1=O)[C@H]2[C@@H]([C@@H]([C@H](O2)CO)O)O	0.43 (0.25,0.73)	1.85E-02	1.37	Maternal third trimester (32–38 weeks gestation) metabolites associated with child asthma/recurrent wheeze status by age 3 in VDAART	3
74	N3-methyluridine	Nucleotide	Pyrimidine Metabolism, Uracil containing	NA	HMDB04813	316991	CN1C(=O)C=CN(C1=O)C2C(C(C(O2)CO)O)O	0.62 (0.44,0.86)	3.46E-02	1.37	NA	NA

No	Metabolites	Super Pathway	Sub Pathway	KEGG	HMDB	PubChem	SMILES	OR (95% CI)	FDR p value	VIP Score	In Silicon Replication	Citation
75	pseudouridine	Nucleotide	Pyrimidine Metabolism, Uracil containing	C02067	HMDB00767	15047	C1=C(C(=O)NC(=O)N1)[C@H]2[C@@H]([C@@H]([C@H](O2)CO)O)O	0.52 (0.33,0.82)	3.67E-02	1.3	The levels of pseudouridine, alpha-N-phenylacetyl-L-glutamine, succinate, L-citrulline, and glycochenodeoxycholate were significantly decreased in asthmatic patients compared with those in the COPD patients	18
76	12-dehydrocholate	Lipid	Secondary Bile Acid Metabolism	C01292	HMDB00400	94235	C[C@H](CCC(=O)O)[C@H]1CC[C@@H]2[C@@]1(C(=O)C[C@H]3[C@H]2[C@@H](C[C@H]4[C@@]3(CC[C@H](C4)O)C)O)C	0.65 (0.48,0.87)	3.29E-02	1.29	NA	NA
77	taurochenodeoxycholic acid 3-sulfate	Lipid	Secondary Bile Acid Metabolism	NA	HMDB02486	52931550	C[C@H](CCC(=O)NCCS(=O)(=O)O)[C@H]1CC[C@@H]2[C@@]1(CC[C@H]3[C@H]2[C@@H](C[C@H]4[C@@]3(CC[C@H](C4)S(=O)(=O)O)C)O)C	0.66 (0.5,0.88)	3.37E-02	1.17	Dose-related systemic effects of inhaled glucocorticoids (GCs)	13
78	2-methylcitrate	Energy	TCA Cycle	C02225	HMDB00379	439681	C[C@H](C(=O)O)[C@](CC(=O)O)(C(=O)O)O	0.48 (0.29,0.78)	2.74E-02	1.15	Metabolites associated with respiratory syncytial virus, increasing the risk of childhood asthma	14
79	malate	Energy	TCA Cycle	C00149	HMDB00156	525	C(C(C(=O)O)O)C(=O)O	1.84 (1.19,2.85)	3.95E-02	1.13	NA	NA
80	alpha-CMBHC glucuronide	Cofactors and Vitamins	Tocopherol Metabolism	NA	NA	NA	NA	0.56 (0.4,0.79)	1.36E-02	1.31	NA	NA
81	indoleacetoylcarnitine*	Amino Acid	Tryptophan Metabolism	NA	NA	NA	NA	0.63 (0.49,0.82)	8.00E-03	1.47	NA	NA
82	xanthurenate	Amino Acid	Tryptophan Metabolism	C02470	HMDB00881	5699	C1=CC2=C(C(=C1)O)NC(=CC2=O)C(=O)O	0.52 (0.34,0.81)	2.91E-02	1.2	Effects on neurotransmitters, catecholeamines, amino acid precursors and metabolites: immune regulation, psychological and behavioural effects.	13

No	Metabolites	Super Pathway	Sub Pathway	KEGG	HMDB	PubChem	SMILES	OR (95% CI)	FDR p value	VIP Score	In Silicon Replication	Citation
83	kynurenine	Amino Acid	Tryptophan Metabolism	C00328	HMDB00684	161166	C1=CC=C(C(=C1)C(=O)C[C@@H](C(=O)O)N)N	0.63 (0.46,0.87)	3.52E-02	1.12	Tryptophan, kynurenine, and neopterin levels were significantly higher in asthmatic children than in healthy controls (p < 0.01; p < 0.01; p = 0.0015 respectively).	16
84	N-acetylkynurenine (2)	Amino Acid	Tryptophan Metabolism	NA	NA	73817779	CC(=O)NC1=CC=CC=C1C(=O)CC(C(=O)O)N	1.64 (1.16,2.32)	3.62E-02	1.6	NA	NA
85	3,4-dihydroxyphenylacetate	Amino Acid	Tyrosine Metabolism	C01161	HMDB01336	547	C1=CC(=C(C=C1CC(=O)O)O)O	0.63 (0.49,0.82)	6.59E-03	1.86	NA	NA
86	3-hydroxyphenylacetatoylcarnitine	Amino Acid	Tyrosine Metabolism	NA	NA	NA	NA	0.69 (0.55,0.88)	2.19E-02	1.44	NA	NA
87	3-methoxytyramine	Amino Acid	Tyrosine Metabolism	C05587	HMDB00022	1669	COC1=C(C=CC(=C1)CCN)O	0.52 (0.34,0.8)	2.56E-02	1.49	NA	NA
88	m-tyramine	Amino Acid	Tyrosine Metabolism	NA	HMDB04989	11492	C1=CC(=CC(=C1)O)CCN	0.56 (0.38,0.82)	2.87E-02	1.17	NA	NA
89	dopamine 4-sulfate	Amino Acid	Tyrosine Metabolism	C13691	HMDB04148	123932	C1=CC(=C(C=C1CCN)O)OS(=O)(=O)O	0.55 (0.36,0.85)	4.44E-02	1.37	NA	NA
90	homoarginine	Amino Acid	Urea cycle; Arginine and Proline Metabolism	C01924	HMDB00670	9085	C(CCN=C(N)N)C[C@@H](C(=O)O)N	0.52 (0.37,0.72)	2.29E-03	1.91	NA	NA

1 Kachroo P, Stewart ID, Kelly RS, et al. Metabolomic profiling reveals extensive adrenal suppression due to inhaled corticosteroid therapy in asthma. *Nature Medicine* 2022; 28(4): 814-22.

2 Mattarucchi E, Baraldi E, Guillou C. Metabolomics applied to urine samples in childhood asthma; differentiation between asthma phenotypes and identification of relevant metabolites. *Biomed Chromatogr* 2012; 26(1): 89-94.

3 Huang M, Kelly RS, Chu SH, et al. Maternal Metabolome in Pregnancy and Childhood Asthma or Recurrent Wheeze in the Vitamin D Antenatal Asthma Reduction Trial. *Metabolites* 2021; 11(2).

4 Kachroo P, Sordillo JE, Lutz SM, et al. Pharmaco-Metabolomics of Inhaled Corticosteroid Response in Individuals with Asthma. *J Pers Med* 2021; 11(11).

5 Reid ES, Papandreou A, Drury S, et al. Advantages and pitfalls of an extended gene panel for investigating complex neurometabolic phenotypes. *Brain* 2016; 139(11): 2844-54.

6 Comhair SA, McDunn J, Bennett C, Fettig J, Erzurum SC, Kalhan SC. Metabolomic Endotype of Asthma. *J Immunol* 2015; 195(2): 643-50.

7 Carraro S, Bozzetto S, Giordano G, et al. Wheezing preschool children with early-onset asthma reveal a specific metabolomic profile. *Pediatr Allergy Immunol* 2018; 29(4): 375-82.

8 Ehrlich SF, Quesenberry CP, Jr., Van Den Eeden SK, Shan J, Ferrara A. Patients diagnosed with diabetes are at increased risk for asthma, chronic obstructive pulmonary disease, pulmonary fibrosis, and pneumonia but not lung cancer. *Diabetes Care* 2010; 33(1): 55-60.

9 Reinke SN, Naz S, Chaleckis R, et al. Urinary metabotype of severe asthma evidences decreased carnitine metabolism independent of oral corticosteroid treatment in the U-BIOPRED study. *European Respiratory Journal* 2022; 59(6): 2101733.

10 Kobayashi H, Amrein K, Lasky-Su JA, Christopher KB. Procalcitonin metabolomics in the critically ill reveal relationships between inflammation intensity and energy utilization pathways. *Scientific Reports* 2021; 11(1): 23194.

11 Barua S, Kuizon S, Junaid MA. Folic acid supplementation in pregnancy and implications in health and disease. *Journal of Biomedical Science* 2014; 21(1): 77.

12 Hallman M, Bry K, Hoppu K, Lappi M, Pohjavuori M. Inositol supplementation in premature infants with respiratory distress syndrome. *N Engl J Med* 1992; 326(19): 1233-9.

No	Metabolites	Super Pathway	Sub Pathway	KEGG	HMDB	PubChem	SMILES	OR (95% CI)	FDR p value	VIP Score	In Silicon Replication	Citation

13 Daley-Yates P, Keppler B, Brealey N, Shabbir S, Singh D, Barnes N. Inhaled glucocorticoid-induced metabolome changes in asthma. *Eur J Endocrinol* 2022; 187(3): 413-27.

14 Stewart CJ, Hasegawa K, Wong MC, et al. Respiratory Syncytial Virus and Rhinovirus Bronchiolitis Are Associated With Distinct Metabolic Pathways. *The Journal of Infectious Diseases* 2018; 217(7): 1160-9.

15 Schjødt MS, Gürdeniz G, Chawes B. The Metabolomics of Childhood Atopic Diseases: A Comprehensive Pathway-Specific Review. *Metabolites* 2020; 10(12).

16 Licari A, Fuchs D, Marseglia G, Ciprandi G. Tryptophan metabolic pathway and neopterin in asthmatic children in clinical practice. *Italian Journal of Pediatrics* 2019; 45(1): 114.

17 Jonkhout N, Tran J, Smith MA, Schonrock N, Mattick JS, Novoa EM. The RNA modification landscape in human disease. *Rna* 2017; 23(12): 1754-69.

18 Ying -L, Yan -GX, Chun -C, Xu -Z, Juan -W, Ting -LT. - Metabolomic Profiling Differences among Asthma, COPD, and Healthy Subjects: A LC-MS-based Metabolomic Analysis. - *Biomedical and Environmental Sciences* 2019; - 32(- 9): - 659.

19 Jin G, Xu M, Zou M, Duan S. The Processing, Gene Regulation, Biological Functions, and Clinical Relevance of N4-Acetylcytidine on RNA: A Systematic Review. *Molecular Therapy - Nucleic Acids* 2020; 20: 13-24.
