# Supplementary material for: Urine metabolomics signature reveals novel determinants of adrenal suppression in children taking inhaled corticosteroids to control asthma symptoms
Source: Immun Inflamm Dis. 2024 Jul 19;12(7):e1315. doi: 10.1002/iid3.1315 (PMC11259003; doi:10.1002/iid3.1315)
Supplement: Supplementary file 3 — Supporting information. [file IID3-12-e1315-s001.pdf]

Supplementary Table 2 Result from Pathway Analysis by MetaboAnalyst 5.0

Pathway	Total number of significant metabolites	Raw p-value	FDR p-value	Impact
Steroid hormone biosynthesis	6	4.25E-06	2.38E-04	0.01
Galactose metabolism	3	9.06E-03	2.54E-01	0.03
Neomycin, kanamycin and gentamicin biosynthesis	1	1.41E-01	1.00E+00	0
Fructose and mannose metabolism	1	1.41E-01	1.00E+00	0.03
Phosphatidylinositol signaling system	1	1.41E-01	1.00E+00	0.04
Histidine metabolism	1	2.29E-01	1.00E+00	0.22
Inositol phosphate metabolism	3	2.63E-01	1.00E+00	0.13
Starch and sucrose metabolism	1	2.63E-01	1.00E+00	0.42
Ascorbate and aldarate metabolism	1	3.68E-01	1.00E+00	0
Taurine and hypotaurine metabolism	1	3.68E-01	1.00E+00	0.43
beta-Alanine metabolism	2	3.71E-01	1.00E+00	0
Primary bile acid biosynthesis	1	4.59E-01	1.00E+00	0.01
Pyruvate metabolism	1	4.59E-01	1.00E+00	0.03
Tyrosine metabolism	2	4.78E-01	1.00E+00	0.02
Pyrimidine metabolism	2	4.78E-01	1.00E+00	0.11
Pentose and glucuronate interconversions	1	5.37E-01	1.00E+00	0.14
Arginine and proline metabolism	2	6.59E-01	1.00E+00	0.02
Citrate cycle (TCA cycle)	1	6.63E-01	1.00E+00	0.04
Glyoxylate and dicarboxylate metabolism	1	7.12E-01	1.00E+00	0
Pantothenate and CoA biosynthesis	1	7.12E-01	1.00E+00	0
Cysteine and methionine metabolism	1	7.12E-01	1.00E+00	0.02
Tryptophan metabolism	1	7.92E-01	1.00E+00	0.09
Glycine, serine and threonine metabolism	1	8.50E-01	1.00E+00	0.03
