# Supplementary material for: Urine metabolomics signature reveals novel determinants of adrenal suppression in children taking inhaled corticosteroids to control asthma symptoms
Source: Immun Inflamm Dis. 2024 Jul 19;12(7):e1315. doi: 10.1002/iid3.1315 (PMC11259003; doi:10.1002/iid3.1315)
Supplement: Supplementary file 4 — Supporting information. [file IID3-12-e1315-s006.pdf]

Supplementary Table 3 List of compounds in significant clusters based on ChemRICH analysis

Compound name	Significant Cluster
4-hydroxyphenylacetylglutamine	Acetylated Peptides
4-hydroxyphenylacetylglycine	Acetylated Peptides
phenylacetylglycine	Acetylated Peptides
phenylacetyl-beta-alanine	Acetylated Peptides
phenylacetyltaurine	Acetylated Peptides
phenylacetylglutamine	Acetylated Peptides
phenylacetylglutamate	Acetylated Peptides
phenylacetylphenylalanine	Acetylated Peptides
phenylacetylalanine	Acetylated Peptides
phenylacetylvaline	Acetylated Peptides
erythronate*	Aminosugar Metabolism
N-acetylglucosaminitol	Aminosugar Metabolism
N-acetylneuraminate	Aminosugar Metabolism
3'-a-sialyl-N-acetyllactosamine	Aminosugar Metabolism
6-sialyl-N-acetyllactosamine	Aminosugar Metabolism
N-acetylglucosaminylasparagine	Aminosugar Metabolism
N-acetylglucosamine/N-acetylgalactosamine	Aminosugar Metabolism
glucuronate	Aminosugar Metabolism
16a-hydroxy DHEA 3-sulfate	Androgenic Steroids
epiandrosterone sulfate	Androgenic Steroids
epiandrosterone glucuronide	Androgenic Steroids
androsterone glucuronide	Androgenic Steroids
etiocholanolone glucuronide	Androgenic Steroids
dehydroepiandrosterone sulfate (DHEA-S)	Androgenic Steroids
androstenediol (3beta,17beta) disulfate (2)	Androgenic Steroids
cortisone	Corticosteroids
cortisol 21-sulfate	Corticosteroids
tetrahydrocortisol	Corticosteroids
cortolone	Corticosteroids
guanidinoacetate	Creatine Metabolism
creatine	Creatine Metabolism
creatinine	Creatine Metabolism
N-methylhydantoin	Creatine Metabolism
phenylalanylglycine	Dipeptide
cyclo(gly-pro)	Dipeptide
prolylglycine	Dipeptide
cyclo(pro-tyr)	Dipeptide
valylglycine	Dipeptide
valylleucine	Dipeptide
glycylleucine	Dipeptide
isoleucylglycine	Dipeptide
methylmalonate (MMA)	Fatty Acid Metabolism (also BCAA Metabolism)
2-methylmalonylcarnitine (C4-DC)	Fatty Acid Metabolism (also BCAA Metabolism)
propionylcarnitine (C3)	Fatty Acid Metabolism (also BCAA Metabolism)
maleate	Fatty Acid, Dicarboxylate

Compound name	Significant Cluster
4-octenedioate	Fatty Acid, Dicarboxylate
2-hydroxyglutarate	Fatty Acid, Dicarboxylate
3-hydroxyadipate*	Fatty Acid, Dicarboxylate
glutarate (C5-DC)	Fatty Acid, Dicarboxylate
2-hydroxyadipate	Fatty Acid, Dicarboxylate
adipate	Fatty Acid, Dicarboxylate
pimelate (C7-DC)	Fatty Acid, Dicarboxylate
suberate (C8-DC)	Fatty Acid, Dicarboxylate
azelate (C9-DC)	Fatty Acid, Dicarboxylate
2-hydroxysebacate	Fatty Acid, Dicarboxylate
sebacate (C10-DC)	Fatty Acid, Dicarboxylate
3-hydroxydodecanedioate*	Fatty Acid, Dicarboxylate
dimethylmalonic acid	Fatty Acid, Dicarboxylate
3-methyladipate	Fatty Acid, Dicarboxylate
3-carboxy-4-methyl-5-propyl-2-furanpropanoate (CMPF	Fatty Acid, Dicarboxylate
3-carboxy-4-methyl-5-pentyl-2-furanpropionate (3-CMP	Fatty Acid, Dicarboxylate
gamma-glutamylglycine	Gamma-glutamyl Amino Acid
gamma-glutamylglutamine	Gamma-glutamyl Amino Acid
gamma-glutamyl-epsilon-lysine	Gamma-glutamyl Amino Acid
gamma-glutamylhistidine	Gamma-glutamyl Amino Acid
gamma-glutamyltyrosine	Gamma-glutamyl Amino Acid
gamma-glutamylphenylalanine	Gamma-glutamyl Amino Acid
gamma-glutamylleucine	Gamma-glutamyl Amino Acid
gamma-glutamylvaline	Gamma-glutamyl Amino Acid
gamma-glutamylisoleucine*	Gamma-glutamyl Amino Acid
gamma-glutamylthreonine	Gamma-glutamyl Amino Acid
hydantoin-5-propionate	Histidine Metabolism
formiminoglutamate	Histidine Metabolism
1-ribosyl-imidazoleacetate*	Histidine Metabolism
trans-urocanate	Histidine Metabolism
cis-urocanate	Histidine Metabolism
4-imidazoleacetate	Histidine Metabolism
imidazole propionate	Histidine Metabolism
histamine	Histidine Metabolism
histidine	Histidine Metabolism
homocarnosine	Histidine Metabolism
carnosine	Histidine Metabolism
imidazole lactate	Histidine Metabolism
N-acetylcarnosine	Histidine Metabolism
N-acetylhistamine	Histidine Metabolism
N-acetyl-1-methylhistidine*	Histidine Metabolism
N-acetylhistidine	Histidine Metabolism
N-acetyl-3-methylhistidine*	Histidine Metabolism
1-methyl-4-imidazoleacetate	Histidine Metabolism
1-methylhistamine	Histidine Metabolism
1-methylhistidine	Histidine Metabolism

Compound name	Significant Cluster
1-methyl-5-imidazoleacetate	Histidine Metabolism
3-methylhistidine	Histidine Metabolism
anserine	Histidine Metabolism
3-methylglutaconate	Leucine, Isoleucine and Valine Metabolism
tigloylglycine	Leucine, Isoleucine and Valine Metabolism
tiglyl carnitine (C5)	Leucine, Isoleucine and Valine Metabolism
3-methylcrotonylglycine	Leucine, Isoleucine and Valine Metabolism
2,3-dimethylsuccinate	Leucine, Isoleucine and Valine Metabolism
beta-hydroxyisovalerate	Leucine, Isoleucine and Valine Metabolism
3-methyl-2-oxobutyrate	Leucine, Isoleucine and Valine Metabolism
isobutyrylglycine (C4)	Leucine, Isoleucine and Valine Metabolism
isobutyrylcarnitine (C4)	Leucine, Isoleucine and Valine Metabolism
N-acetylvaline	Leucine, Isoleucine and Valine Metabolism
N-carbamoylvaline	Leucine, Isoleucine and Valine Metabolism
alpha-hydroxyisovalerate	Leucine, Isoleucine and Valine Metabolism
4-methyl-2-oxopentanoate	Leucine, Isoleucine and Valine Metabolism
isovalerylglycine	Leucine, Isoleucine and Valine Metabolism
isovalerylcarnitine (C5)	Leucine, Isoleucine and Valine Metabolism
N-succinyl-leucine	Leucine, Isoleucine and Valine Metabolism
leucine	Leucine, Isoleucine and Valine Metabolism
N-methylleucine	Leucine, Isoleucine and Valine Metabolism
N-acetylleucine	Leucine, Isoleucine and Valine Metabolism
valine	Leucine, Isoleucine and Valine Metabolism
1-carboxyethylvaline	Leucine, Isoleucine and Valine Metabolism
methylsuccinate	Leucine, Isoleucine and Valine Metabolism
3-methylglutarylcarnitine (2)	Leucine, Isoleucine and Valine Metabolism
3-hydroxyisobutyrate	Leucine, Isoleucine and Valine Metabolism
ethylmalonate	Leucine, Isoleucine and Valine Metabolism
2,3-dihydroxy-3-methylvalerate	Leucine, Isoleucine and Valine Metabolism
3-methyl-2-oxovalerate	Leucine, Isoleucine and Valine Metabolism
2-methylbutyrylglycine (C5)	Leucine, Isoleucine and Valine Metabolism
2-methylbutyrylcarnitine (C5)	Leucine, Isoleucine and Valine Metabolism
N-acetylisoleucine	Leucine, Isoleucine and Valine Metabolism
N-succinyl-isoleucine	Leucine, Isoleucine and Valine Metabolism
3-hydroxy-2-ethylpropionate	Leucine, Isoleucine and Valine Metabolism
isoleucine	Leucine, Isoleucine and Valine Metabolism
1-carboxyethylisoleucine	Leucine, Isoleucine and Valine Metabolism
1-carboxyethylleucine	Leucine, Isoleucine and Valine Metabolism
2-oxoadipate	Lysine Metabolism
5-hydroxylysine	Lysine Metabolism
2-aminoadipate	Lysine Metabolism
5-(galactosylhydroxy)-L-lysine	Lysine Metabolism
5-aminovalerate	Lysine Metabolism
lysine	Lysine Metabolism
fructosyllysine	Lysine Metabolism
6-oxopiperidine-2-carboxylate	Lysine Metabolism

Compound name	Significant Cluster
pipecolate	Lysine Metabolism
N2,N6-diacetyllysine	Lysine Metabolism
N-acetyl-cadaverine	Lysine Metabolism
N6-acetyllysine	Lysine Metabolism
N2-acetyllysine	Lysine Metabolism
N2-acetyl,N6-methyllysine	Lysine Metabolism
N6-carboxyethyllysine	Lysine Metabolism
N6,N6-dimethyllysine	Lysine Metabolism
N6-methyllysine	Lysine Metabolism
glutarylcarnitine (C5-DC)	Lysine Metabolism
N,N,N-trimethyl-5-aminovalerate	Lysine Metabolism
N6,N6,N6-trimethyllysine	Lysine Metabolism
taurine	Methionine, Cysteine, SAM and Taurine Metabolism
hypotaurine	Methionine, Cysteine, SAM and Taurine Metabolism
cystathionine	Methionine, Cysteine, SAM and Taurine Metabolism
cysteine	Methionine, Cysteine, SAM and Taurine Metabolism
lanthionine	Methionine, Cysteine, SAM and Taurine Metabolism
cysteine s-sulfate	Methionine, Cysteine, SAM and Taurine Metabolism
cystine	Methionine, Cysteine, SAM and Taurine Metabolism
S-adenosylhomocysteine (SAH)	Methionine, Cysteine, SAM and Taurine Metabolism
N-acetylmethionine sulfoxide	Methionine, Cysteine, SAM and Taurine Metabolism
N-acetylmethionine	Methionine, Cysteine, SAM and Taurine Metabolism
N-acetyltaurine	Methionine, Cysteine, SAM and Taurine Metabolism
N-acetylcysteine	Methionine, Cysteine, SAM and Taurine Metabolism
N-methyltaurine	Methionine, Cysteine, SAM and Taurine Metabolism
methionine sulfone	Methionine, Cysteine, SAM and Taurine Metabolism
S-methylcysteine sulfoxide	Methionine, Cysteine, SAM and Taurine Metabolism
methionine sulfoxide	Methionine, Cysteine, SAM and Taurine Metabolism
5-methylthioribose	Methionine, Cysteine, SAM and Taurine Metabolism
methionine	Methionine, Cysteine, SAM and Taurine Metabolism
N-formylmethionine	Methionine, Cysteine, SAM and Taurine Metabolism
S-adenosylmethionine (SAM)	Methionine, Cysteine, SAM and Taurine Metabolism
isoputreanine	Polyamine Metabolism
spermidine	Polyamine Metabolism
diacetylspermidine*	Polyamine Metabolism
4-acetamidobutanoate	Polyamine Metabolism
N-acetylputrescine	Polyamine Metabolism
acetylagmatine	Polyamine Metabolism
acisoga	Polyamine Metabolism
N-acetyl-isoputreanine	Polyamine Metabolism
N1,N12-diacetylspermine	Polyamine Metabolism
5-methylthioadenosine (MTA)	Polyamine Metabolism
allantoic acid	Purine Metabolism, (Hypo)Xanthine/Inosine containing
uric acid ribonucleoside*	Purine Metabolism, (Hypo)Xanthine/Inosine containing
allantoin	Purine Metabolism, (Hypo)Xanthine/Inosine containing
urate	Purine Metabolism, (Hypo)Xanthine/Inosine containing

Compound name	Significant Cluster
hypoxanthine	Purine Metabolism, (Hypo)Xanthine/Inosine containing
xanthine	Purine Metabolism, (Hypo)Xanthine/Inosine containing
xanthosine	Purine Metabolism, (Hypo)Xanthine/Inosine containing
N1-methylinosine	Purine Metabolism, (Hypo)Xanthine/Inosine containing
1-methylhypoxanthine	Purine Metabolism, (Hypo)Xanthine/Inosine containing
N2-methylguanosine	Purine Metabolism, Guanine containing
1-methylguanosine	Purine Metabolism, Guanine containing
1-methylguanine	Purine Metabolism, Guanine containing
cytosine	Pyrimidine Metabolism, Cytidine containing
cytidine	Pyrimidine Metabolism, Cytidine containing
N4-acetylcytidine	Pyrimidine Metabolism, Cytidine containing
3-methylcytidine	Pyrimidine Metabolism, Cytidine containing
2'-O-methylcytidine	Pyrimidine Metabolism, Cytidine containing
4-ureidobutyrate	Pyrimidine Metabolism, Uracil containing
3-ureidopropionate	Pyrimidine Metabolism, Uracil containing
pseudouridine	Pyrimidine Metabolism, Uracil containing
3-(3-amino-3-carboxypropyl)uridine*	Pyrimidine Metabolism, Uracil containing
uridine	Pyrimidine Metabolism, Uracil containing
uracil	Pyrimidine Metabolism, Uracil containing
5,6-dihydrouridine	Pyrimidine Metabolism, Uracil containing
5,6-dihydrouracil	Pyrimidine Metabolism, Uracil containing
N-acetyl-beta-alanine	Pyrimidine Metabolism, Uracil containing
5-methyluridine (ribothymidine)	Pyrimidine Metabolism, Uracil containing
N3-methyluridine	Pyrimidine Metabolism, Uracil containing
2'-O-methyluridine	Pyrimidine Metabolism, Uracil containing
glycolithocholate sulfate*	Secondary Bile Acid Metabolism
glycoursodeoxycholate	Secondary Bile Acid Metabolism
glycocholenate sulfate*	Secondary Bile Acid Metabolism
glycohyocholate	Secondary Bile Acid Metabolism
taurolithocholate 3-sulfate	Secondary Bile Acid Metabolism
taurochenodeoxycholic acid 3-sulfate	Secondary Bile Acid Metabolism
taurocholenate sulfate*	Secondary Bile Acid Metabolism
12-dehydrocholate	Secondary Bile Acid Metabolism
ursocholate	Secondary Bile Acid Metabolism
trans-aconitate	TCA Cycle
fumarate	TCA Cycle
citrate	TCA Cycle
carboxysuccinate	TCA Cycle
malate	TCA Cycle
isocitrate	TCA Cycle
tricarballylate	TCA Cycle
alpha-ketoglutarate	TCA Cycle
succinate	TCA Cycle
isocitric lactone	TCA Cycle
mesaconate (methylfumarate)	TCA Cycle
2-methylcitrate	TCA Cycle

Compound name	Significant Cluster
succinylcarnitine (C4-DC)	TCA Cycle
3-hydroxykynurenine	Tryptophan Metabolism
3-hydroxyanthranilate	Tryptophan Metabolism
5-hydroxypicolinic acid	Tryptophan Metabolism
xanthurenate	Tryptophan Metabolism
6-bromotryptophan	Tryptophan Metabolism
5-hydroxyindoleacetate	Tryptophan Metabolism
serotonin	Tryptophan Metabolism
5-hydroxyindole sulfate	Tryptophan Metabolism
kynurenine	Tryptophan Metabolism
anthranilate	Tryptophan Metabolism
N-formylanthranilic acid	Tryptophan Metabolism
C-glycosyltryptophan	Tryptophan Metabolism
indole-3-carboxylate	Tryptophan Metabolism
indoleacetylglutamine	Tryptophan Metabolism
indoleacetylglycine	Tryptophan Metabolism
indoleacetate	Tryptophan Metabolism
indolelactate	Tryptophan Metabolism
indolepropionylglycine	Tryptophan Metabolism
tryptamine	Tryptophan Metabolism
tryptophan	Tryptophan Metabolism
3-indoxyl sulfate	Tryptophan Metabolism
indoxyl glucuronide	Tryptophan Metabolism
kynurenate	Tryptophan Metabolism
picolinate	Tryptophan Metabolism
N-acetylkynurenine (2)	Tryptophan Metabolism
N-acetyltryptophan	Tryptophan Metabolism
8-methoxykynurenate	Tryptophan Metabolism
5-hydroxymethyl-2-furoic acid	Tyrosine Metabolism
3,4-dihydroxyphenylacetate	Tyrosine Metabolism
3,4-dihydroxyphenylacetate sulfate	Tyrosine Metabolism
dopamine 4-sulfate	Tyrosine Metabolism
dopamine 3-O-sulfate	Tyrosine Metabolism
gentisate	Tyrosine Metabolism
m-tyramine	Tyrosine Metabolism
4-hydroxycinnamate sulfate	Tyrosine Metabolism
4-hydroxyphenylpyruvate	Tyrosine Metabolism
3-(4-hydroxyphenyl)lactate (HPLA)	Tyrosine Metabolism
tyramine	Tyrosine Metabolism
tyramine O-sulfate	Tyrosine Metabolism
tyrosine	Tyrosine Metabolism
o-Tyrosine	Tyrosine Metabolism
catechol glucuronide	Tyrosine Metabolism
phenol sulfate	Tyrosine Metabolism
phenol glucuronide	Tyrosine Metabolism
N-acetyltyrosine	Tyrosine Metabolism

Compound name	Significant Cluster
1-carboxyethyltyrosine	Tyrosine Metabolism
p-cresol glucuronide*	Tyrosine Metabolism
vanillylmandelate (VMA)	Tyrosine Metabolism
homovanillate (HVA)	Tyrosine Metabolism
3-methoxytyrosine	Tyrosine Metabolism
vanillactate	Tyrosine Metabolism
3-methoxytyramine	Tyrosine Metabolism
3-methoxytyramine sulfate	Tyrosine Metabolism
