# Supplementary material for: Urine metabolomics signature reveals novel determinants of adrenal suppression in children taking inhaled corticosteroids to control asthma symptoms
Source: Immun Inflamm Dis. 2024 Jul 19;12(7):e1315. doi: 10.1002/iid3.1315 (PMC11259003; doi:10.1002/iid3.1315)
Supplement: Supplementary file 5 — Supporting information. [file IID3-12-e1315-s003.pdf]

Supplementary Table 4 List of significant metabolites associated with 24h-urinary cortisol level in CAMP (raw p-value of 0.05)

Metabolite	HMDB	KEGG	PubChem	Super pathway	Sub pathway	raw p-value
alpha-hydroxybutyrate	HMDB00008	C05984	440864	Amino Acid	Glutathione Metabolism	1.80E-02
dimethylglycine	HMDB00092	C01026	673	Amino Acid	Glycine, Serine and Threonine Metabolism	3.49E-02
urocanic acid	HMDB00301	C00785	736715	Amino Acid	Histidine Metabolism	9.32E-03
cystathionine	HMDB00099	C02291	439258	Amino Acid	Methionine, Cysteine, SAM and Taurine Metabolism	2.41E-02
sorbitol	HMDB00247	C00794	5780	Carbohydrate	Fructose, Mannose and Galactose Metabolism	4.51E-02
biliverdin	HMDB01008	C00500	5353439	Cofactors and Vitamins	Hemoglobin and Porphyrin Metabolism	9.85E-04
11-HETE	HMDB04682	C14780	5283168	Lipid	Arachidonic acid metabolism	4.92E-02
C36:4 DAG	HMDB07248*	C00165	9543729	Lipid	Diacylglycerol	3.47E-02
C36:3 DAG	HMDB07219*	C00165	9543722	Lipid	Diacylglycerol	4.93E-02
C12:1 carnitine	HMDB13326	NA	53481671	Lipid	Fatty Acid Metabolism (Acyl Carnitine, Monounsaturated)	2.52E-02
C18:2 carnitine	HMDB06469	NA	6450015	Lipid	Fatty Acid Metabolism (Acyl Carnitine, Polyunsaturated)	5.00E-02
C2 carnitine	HMDB00201	C02571	1	Lipid	Fatty Acid Metabolism (Acyl Carnitine, Short Chain)	2.41E-02
5-dodecenoate	HMDB00529	NA	5312377	Lipid	Fatty Acid Synthesis	4.15E-02
malonate	HMDB00691	C04025	867	Lipid	Fatty Acid Synthesis	4.53E-02
alpha-glycerophosphate	HMDB00126	C00093	439162	Lipid	Glycerolipid Metabolism	1.65E-02
docosahexaenoate	HMDB02183	C06429	445580	Lipid	Long Chain Polyunsaturated Fatty Acid (n3 and n6)	6.87E-03
docosapentaenoate	HMDB01976	NA	6441454	Lipid	Long Chain Polyunsaturated Fatty Acid (n3 and n6)	7.68E-03
arachidonate	HMDB01043	C00219	444899	Lipid	Long Chain Polyunsaturated Fatty Acid (n3 and n6)	2.02E-02
eicosadienoate	HMDB05060	C16525	6439848	Lipid	Long Chain Polyunsaturated Fatty Acid (n3 and n6)	2.59E-02
adrenate	HMDB02226	C16527	5497181	Lipid	Long Chain Polyunsaturated Fatty Acid (n3 and n6)	2.82E-02

Metabolite	HMDB	KEGG	PubChem	Super pathway	Sub pathway	raw p-value
eicosatrienoate	HMDB02925	C03242	5280581	Lipid	Long Chain Polyunsaturated Fatty Acid (n3 and n6)	3.16E-02
C36:3 PS plasmalogen	NA	NA	NA	Lipid	Pregnenolone Steroids	2.93E-02
taurochenodeoxycholate	HMDB00951	C05465	387316	Lipid	Primary Bile Acid Metabolism	6.12E-04
chenodeoxycholate	HMDB00518	C02528	10133	Lipid	Primary Bile Acid Metabolism	1.00E-02
glycochenodeoxycholate	HMDB00637	C05466	22833540	Lipid	Primary Bile Acid Metabolism	1.32E-02
taurodeoxycholate	HMDB00896	C05463	2733768	Lipid	Secondary Bile Acid Metabolism	1.12E-03
taurodeoxycholate/taurochenodeoxycholate	HMDB00896*	C05463	2733768	Lipid	Secondary Bile Acid Metabolism	4.10E-02
C58:7 TAG	HMDB05471*	C00422	9545051	NA	NA	3.71E-03
C18:3 CE	HMDB10370*	C02530	6436907	NA	NA	4.73E-03
C56:4 TAG	HMDB05398*	C00422	9544519	NA	NA	4.75E-03
8-HDoHE	HMDB60051	NA	131769810	NA	NA	1.03E-02
13-HDoHE	HMDB60043	NA	131769803	NA	NA	1.28E-02
16-HDoHE/17-HDoHE	HMDB60047*	NA	131769806	NA	NA	1.38E-02
C43:1 TAG	HMDB42098*	NA	131753244	NA	NA	2.26E-02
7-HDoHE	HMDB60050	NA	131769809	NA	NA	2.50E-02
C46:0 TAG	HMDB10411*	NA	545606	NA	NA	2.57E-02
C52:4 TAG	HMDB05363*	C00422	9544125	NA	NA	3.16E-02
11-HDoHE	HMDB60040	NA	131769802	NA	NA	3.29E-02
C54:4 TAG	HMDB05370*	C00422	9544279	NA	NA	4.43E-02
C54:5 TAG	HMDB05385*	C00422	9544319	NA	NA	4.46E-02
C44:0 TAG	HMDB42063*	NA	97045147	NA	NA	4.53E-02
20-HDoHE	HMDB60048	NA	131769807	NA	NA	4.56E-02
C56:5 TAG	HMDB05406*	C00422	9544577	NA	NA	4.58E-02
2-methylguanosine	HMDB05862	NA	NA	Nucleotide	Purine Metabolism, Guanine containing	4.03E-02
uracil	HMDB00300	C00106	1174	Nucleotide	Pyrimidine Metabolism, Uracil containing	3.96E-02
levulinate	HMDB00720	NA	11579	Xenobiotics	Food Component/Plant	2.14E-02
