# Supplementary material for: Urine metabolomics signature reveals novel determinants of adrenal suppression in children taking inhaled corticosteroids to control asthma symptoms
Source: Immun Inflamm Dis. 2024 Jul 19;12(7):e1315. doi: 10.1002/iid3.1315 (PMC11259003; doi:10.1002/iid3.1315)
Supplement: Supplementary file 6 — Supporting information. [file IID3-12-e1315-s004.pdf]

Supplementary Table 5 List of significant metabolites associated with baseline, 30 min and 60 min plasma cortisol level in CAMP (raw p-value of 0.05)

Metabolite	HMDB	KEGG	PubChem	Super pathway	Sub pathway	raw p-value
						**Baseline plasma cortisol**
17-Methylstearate	HMDB61710	NA	3083779			1.50E-02
urobilin	HMDB04159	C05793	5280818	Cofactors and Vitamins	Hemoglobin and Porphyrin Metabolism	1.72E-02
C36:5 PC plasmalogen	HMDB11220*	NA	24779388	Lipid	Plasmalogen	2.76E-02
C14:0 CE	HMDB06725	NA	99486			3.25E-02
C22:5 LPC	HMDB10403*	C04230	53480475	Lipid	Lysophospholipid	3.27E-02
C16:1 CE	HMDB00658*	NA	22833543			4.14E-02
cytidine	HMDB00089	C00475	6253	Nucleotide	Pyrimidine Metabolism, Cytidine containing	4.42E-02
stearate	HMDB00827	C01530	5281	Lipid	Long Chain Saturated Fatty Acid	4.60E-02
						**30 min plasma cortisol**
C22:5 LPC	HMDB0010403	C04230	53480475	Lipid	Lysophospholipid	6.19E-04
urobilin	HMDB0004159	C05793	5280818	Cofactors and Vitamins	Hemoglobin and Porphyrin Metabolism	1.77E-03
stearate	HMDB0000827	C01530	5281	Lipid	Long Chain Saturated Fatty Acid	1.06E-02
C14:0 CE	HMDB0006725	NA	99486			1.11E-02
N-acetylalanine	HMDB0000766	NA	88064	Amino Acid	Alanine and Aspartate Metabolism	2.23E-02
acetaminophen	HMDB0001859	C06804	1983	Xenobiotics	Drug - Analgesics, Anesthetics	2.42E-02
DMGV	HMDB0240212	NA	101535539			2.55E-02
nonadecanoate	HMDB0000772	C16535	12591	Lipid	Long Chain Saturated Fatty Acid	2.72E-02
12-HETE/8-HETE	HMDB0006111	C14777	5312983	Lipid	Eicosanoid	2.79E-02
methylmalonate	HMDB0000202	C02170	487	Lipid	Fatty Acid Metabolism (also BCAA Metabolism)	2.97E-02

Metabolite	HMDB	KEGG	PubChem	Super pathway	Sub pathway	raw p-value
trigonelline	HMDB0000875	C01004	5570	Cofactors and Vitamins	Nicotinate and Nicotinamide Metabolism	3.37E-02
17-Methylstearate	HMDB0061710	NA	3083779			3.52E-02
kynurenine	HMDB0000684	C00328	161166	Amino Acid	Tryptophan Metabolism	4.86E-02
**60 min plasma cortisol**						
DMGV	HMDB0240212	NA	101535539			2.63E-04
acetaminophen	HMDB0001859	C06804	1983	Xenobiotics	Drug - Analgesics, Anesthetics	1.32E-02
PGE2	HMDB0001220	C00584	5283116	Lipid	Eicosanoid	1.39E-02
eicosatrienoate	HMDB0002925	C03242	5280581	Lipid	Long Chain Polyunsaturated Fatty Acid (n3 and n6)	1.45E-02
C38:3 PE plasmalogen	HMDB0011384	NA	52925088			2.31E-02
C40:7 PE plasmalogen	HMDB0011394	NA	42607458	Lipid	Plasmalogen	2.37E-02
PGE1	HMDB0001442	C04741	5280723			2.69E-02
dimethylurate	HMDB0001857	NA	70346	Xenobiotics	Xanthine Metabolism	2.87E-02
RvD2	HMDB0002294	NA	16061136			2.90E-02
saccharin	HMDB0029723	C12283	5143	Xenobiotics	Food Component/Plant	2.93E-02
12-HETE/8-HETE	HMDB0006111	C14777	5312983	Lipid	Eicosanoid	3.15E-02
hexose monophosphate	HMDB0000124	C00085	69507			3.23E-02
C36:5 PC plasmalogen-A	HMDB0011221	NA	53480687			3.34E-02
C20:5 CE	HMDB06731	NA	10372299			3.74E-02
PGF2	HMDB0001483	C02314	5280506			3.98E-02
5-hydroxytryptophol	HMDB0001855	NA	9061			4.27E-02
palmitate	HMDB0000220	C00249	985	Lipid	Long Chain Saturated Fatty Acid	4.45E-02

**Metabolite**	**HMDB**	**KEGG**	**PubChem**	**Super pathway**	**Sub pathway**	**raw p-value**
C40:10 PC	HMDB0008511	C00157	52923375			4.53E-02
9-HETE	HMDB10222	NA	5312979			4.78E-02
pseudouridine	HMDB0000767	C02067	15047	Nucleotide	Pyrimidine Metabolism, Uracil containing	4.91E-02
eicosadienoate	HMDB0005060	C16525	6439848	Lipid	Long Chain Polyunsaturated Fatty Acid (n3 and n6)	4.95E-02
